# The reporting phenotype of penile fracture after collagenase *clostridium* histolyticum for Peyronie’s disease: an indication-restricted FAERS study

**DOI:** 10.3389/fphar.2026.1965417

**Published:** 2026-10-14

**Authors:** Xin Qian, Can Ran, Yangyang Gong, Xiang Cai, Linlin Xiang, Mingdong Jiang, Hui Tian, Yougang Feng, Hongjian Liu

**Affiliations:** Department of Urology, Suining Central Hospital, Suining, China

**Keywords:** collagenase *clostridium* histolyticum, FDA adverse event reporting system, penile fracture, Peyronie’s disease, pharmacovigilance, quantitative bias analysis, reporting phenotype, therapy-start-to-event interval

## Abstract

**Background:**

Corporal rupture and severe penile injury are established complications of collagenase *clostridium* histolyticum (CCH) treatment for Peyronie’s disease. How spontaneous reports jointly encode injury, management, hospitalization, timing, and procedural or product-use details remains unclear.

**Methods:**

We analyzed quarterly FDA Adverse Event Reporting System files from 2014Q1 through 2025Q4. We retained the latest, deletion-list-aware report version when CCH was the primary suspect for a strict Peyronie’s disease indication. We compared a fracture-coded phenotype (F1) with a mutually exclusive non-fracture local-injury phenotype (I1). Raw report proportions with exact confidence intervals were the primary descriptive results. Firth models provided secondary control for reporting period, healthcare professional reporter, and manufacturer origin. Analyses also considered surgery/repair (M1), hospitalization, a heterogeneous procedural/product-use summary (P1), and a report-level therapy-start-to-event interval.

**Results:**

Of 3,750 latest-version strict reports, 3,658 named CCH as the sole primary suspect. The active comparison included 445 F1 and 834 I1 reports. M1 was recorded in 194/445 F1 and 30/834 I1 reports. Hospitalization was recorded in 2.5% of I1 reports without M1, 8.0% of F1 reports without M1, and 26.3% of F1 reports with M1. P1 was recorded in 57/445 F1 reports (12.8%, exact 95% CI: 9.8%–16.3%) and 171/834 I1 reports (20.5%, 17.8%–23.4%); the crude odds ratio was 0.57 (exact 95% CI: 0.40–0.80), and the adjusted reporting odds ratio was 0.50 (95% CI: 0.35–0.71). The subdomains were directionally discordant. After 20 P1-negative F1 reports were reclassified, the balanced adjusted 95% CI included 1; reclassification of 35 reports equalized the crude proportions. A therapy-start-to-event interval was available for 91/445 F1 reports (median 14 days, IQR 1–27), and entry into this date-complete subset was non-random.

**Conclusion:**

Fracture-coded FAERS reports after CCH form a clinically coherent but heterogeneous phenotype, often accompanied by surgery/repair or hospitalization codes. P1 is a reporting-process summary rather than a unitary clinical construct: its inverse aggregate association concealed discordant components, was sensitive to modest differential under-recording, and does not indicate protection. The study refines the reporting phenotype of a known serious risk without estimating incidence, comparative safety, or causation.

## 1 Introduction

Peyronie’s disease is an acquired fibrotic disorder of the tunica albuginea. It can cause penile curvature, shortening, pain, erectile dysfunction, and impaired sexual function. European and international guidance recommends phase-specific assessment, careful documentation of deformity, and treatment aligned with each patient’s functional goals and preferred benefit–harm balance (Salonia et al., 2025; Chung et al., 2026). Prevalence estimates depend strongly on case definition, with physician-confirmed disease less frequent than symptom-defined probable disease (Stuntz et al., 2016). Clinical presentation and the course across active and stable phases are also heterogeneous (Di Maida et al., 2020; Paulis et al., 2024).

Beyond penile anatomy, the burden of Peyronie’s disease includes substantial anxiety and depression in clinical series (Paulis and Paulis, 2026). Nationwide data also indicate an increased subsequent risk of depression (Lindhardt-Mejlholm et al., 2025). Female partners may experience related sexual and relationship difficulties (Farrell et al., 2020). Population-level evidence also suggests a higher likelihood of relationship separation (Henningsohn et al., 2024). These effects arise in the setting of a still incompletely understood fibrotic wound-healing process involving transforming growth factor-β and other pro-fibrotic pathways (Mitsui et al., 2023).

Treatment options include observation, mechanical therapy, intralesional treatment, and surgery. The American Urological Association guideline places these options within an evidence-based framework that also recognizes individual values and goals (Nehra et al., 2015). Collagenase *clostridium* histolyticum (CCH) was the first pharmacological treatment supported by two large phase 3 randomized trials, which improved curvature and symptom bother (Gelbard et al., 2013). Open-label evidence extended these findings (Levine et al., 2014), and a meta-analysis of prospective studies supports an average improvement in curvature (Zhang et al., 2022). A Cochrane review nevertheless found continuing uncertainty across nonsurgical therapies and patient-important outcomes (Rosenberg et al., 2023). CCH is, therefore, one component of individualized nonsurgical care rather than a universally preferred treatment (Pinkhasov et al., 2024).

Local bruising, swelling, and pain are expected after CCH, but corporal rupture and severe penile hematoma are established serious risks. In a pooled analysis of six clinical studies, most treatment-related events were non-serious. Serious corporal rupture and hematoma were uncommon but clinically important (Carson et al., 2015). Post-CCH injury is not a uniform clinical event, and its recognition and management depend on presentation (Furtado et al., 2024). Practice surveys document substantial variation in the diagnosis and management of suspected rupture (Yafi et al., 2016). Rupture may also be recognized beyond the immediate post-injection period (Beilan et al., 2017), and management reviews treat CCH-associated fracture as a distinct clinical setting (Hughes et al., 2021). Some suspected fractures have been managed conservatively without evident deterioration in erectile function (Sharma et al., 2020). Regulatory summaries, trials, and clinical cohorts establish the risk and provide patient-level detail. They do not show how spontaneous reports represent injury, management, hospitalization, timing, and procedural details together.

A conventional disproportionality screen could rediscover this labeled event while overlooking the structure of the reports that generated it. We, therefore, examined US Food and Drug Administration Adverse Event Reporting System (FAERS) reports in which CCH was the primary suspect for Peyronie’s disease. The analysis combined a fracture-coded phenotype, an active non-fracture local-injury comparator, surgery/repair and hospitalization attributes, and a heterogeneous procedural/product-use reporting summary. We characterized the injury-by-management phenotype, examined a restricted coding order, and described the selected therapy-start-to-event subset. We also quantified sensitivity to differential under-recording and compared the findings with non-poolable regulatory, trial, and cohort evidence. The objective was to refine the reporting phenotype and define its inferential limits, not to estimate incidence, comparative safety, or causation.

## 2 Materials and methods

### 2.1 Study design, data source, and reporting framework

We conducted a retrospective, report-level pharmacovigilance study of the FDA Adverse Event Reporting System (FAERS). The analysis used ASCII quarterly files from 2014Q1 through 2025Q4. FAERS receives mandatory manufacturer reports and voluntary reports from healthcare professionals and consumers. The quarterly files do not provide a denominator of treated patients (U.S. Food and Drug Administration, 2026). Reaction and indication terms use the Medical Dictionary for Regulatory Activities (MedDRA) hierarchy (MedDRA Maintenance and Support Services Organization, 2026). Reporting followed READUS-PV (Fusaroli et al., 2024a; Fusaroli et al., 2024b) and relevant RECORD principles for routinely collected observational data (Benchimol et al., 2015).

A dated protocol and a FAERS-specific statistical analysis plan specified the phenotypes, active comparator, P1 components, core covariates, sensitivity analyses, and interpretation limits. The protocol and plan were frozen before the corresponding analyses. After completing the initial ladder and P1 models, a dated amendment prospectively specified the categorical and linear hospitalization analyses and the deterministic covariate-balanced tipping path before we ran those added analyses. The dated protocol and SAP/amendment are provided as Supplementary Files S2, S3.

### 2.2 Report reconstruction, product identification, and strict indication restriction

We scanned all 48 quarterly archives for candidate CASEIDs containing a strict lexical variant of the MedDRA concept “Peyronie’s disease.” The term “penile curvature” alone did not qualify. We then rescanned every DEMO version of each candidate CASEID within the study window. For each candidate, we retained the highest CASEVERSION, using FDA receipt date and PRIMARYID as sequential tie-breakers. The retained version still had to contain the strict indication. CASEIDs appearing in FDA-deleted-case files were excluded at this stage.

CCH exposure was identified from normalized drug names, active-ingredient fields, and explicit XIAFLEX or XIAPEX product names. Eligibility required a CCH drug sequence coded as primary suspect and linked to the strict Peyronie’s disease indication. We then enumerated every primary-suspect drug sequence in the retained report. No eligible CCH-primary-suspect report contained a second primary-suspect product; thus, “sole primary suspect” describes the observed final cohort rather than an additional outcome-dependent exclusion. Reports listing CCH only as secondary suspect, concomitant, or interacting were excluded. Supplementary Table S1 summarizes product and indication linkage, drug-role restrictions, and report-version rules.

### 2.3 Injury, management, procedural, and severity phenotypes

The fracture-coded phenotype (F1) required the exact MedDRA preferred term “Fracture of penis.” F1 was a coding phenotype, not an adjudicated corporal rupture. The mutually exclusive active comparator was the non-fracture local-injury phenotype (I1). I1 required the absence of F1 and at least one prespecified penile or genital injury term. These terms covered hematoma, hemorrhage, contusion, swelling, edema, pain, injury, or laceration, as detailed in Supplementary Table S1.

The surgery/repair phenotype (M1) required “corpora cavernosa surgery,” “penile operation,” or “penile surgery.” Hospitalization was defined by the FAERS outcome code for hospitalization or prolonged hospitalization. M1 and hospitalization were co-occurring report attributes, without an assumed temporal or causal link to a specific reaction term.

The procedural/product-use issue summary (P1) contained 12 frozen terms. It was prespecified as a high-sensitivity indicator that a report contained any procedural or product-use context, not as a single clinical exposure or mechanism. Four prespecified, non-exclusive domains comprised technique/site, schedule/dose/duration, treatment nonadherence, and unapproved/intentional/general use (Supplementary Table S1). Because these domains are clinically heterogeneous, their observed frequencies were foregrounded, and the aggregate P1 estimate was interpreted only as a reporting-process summary. The complete injury-by-management phenotype cross-classified F1 or I1 by M1 status. A restricted ordering then defined L0 as I1 without M1, L1 as F1 without M1, and L2 as F1 with M1. I1 reports with M1 were described separately and excluded because a surgery-coded report could not plausibly occupy the lowest rung. The resulting construct described a restricted coding hierarchy rather than within-person clinical progression.

### 2.4 Reporting-process variables, concomitant drugs, and missing data

Report periods were grouped as 2014–2017, 2018–2021, and 2022–2025. The healthcare professional indicator included physicians, pharmacists, nurses, other healthcare professionals, and other coded professional categories. Manufacturer origin was derived from the report type and manufacturer sender fields. The prespecified core adjustment set comprised reporting period and reporter type, which were strong reporting-process determinants, plus manufacturer origin to preserve a common model specification despite its limited variation. Its purpose was secondary control of measured reporting-process differences, not causal effect estimation. Literature source and US origin were not included in the core model because they were sparse or highly imbalanced reporting-channel markers and could make the primary specification dependent on a few cells; they were included, with report-richness measures, in a prespecified sensitivity model. Prespecified concomitant-drug flags covered anticoagulants, antiplatelets, and phosphodiesterase type 5 inhibitors.

Age was converted to years using the reported unit, and values reported outside the range of 0–120 years were set to missing. Prespecified categories were <65, 65–74, ≥75, and missing age. Missing values were not multiply imputed, and age was omitted from primary adjustment because it was unavailable in many reports. Complete-case and missing-category age models were used as sensitivity analyses. Preferred-term and drug counts were included only in report-richness sensitivity analyses and the TTO-availability model.

### 2.5 Descriptive analyses

Categorical variables were summarized as counts and report proportions, while age and the therapy-start-to-event interval were summarized by medians and interquartile ranges (IQRs). Table 1 uses standardized mean differences rather than null-hypothesis tests to describe F1–I1 differences. Exact binomial 95% confidence intervals were calculated for key cell-specific proportions. The unadjusted F1-versus-I1 P1 odds ratio and exact 95% confidence interval were obtained using a two-sided Fisher exact test. Annual volume and completeness were treated as reporting-process measures, not incidence trends.

**TABLE 1:** Characteristics of deletion-list-aware F1 and I1 reports.

Section	Characteristic	Level	F1 fracture-coded phenotype (n = 445)	I1 non-fracture local-injury phenotype (n = 834)	SMD
Demographic information	Age, years	Median [IQR]; available n/N	53.0 [48.0, 60.0]; 213/445	57.0 [50.0, 62.0]; 465/834	−0.264
	Age group	<65	192 (43.1%)	398 (47.7%)	−0.092
	Age group	65–74	19 (4.3%)	63 (7.6%)	−0.140
	Age group	≥75	2 (0.4%)	4 (0.5%)	−0.004
	Age group	Missing	232 (52.1%)	369 (44.2%)	0.158
Reporting process	Healthcare-professional reporter	Yes	228 (51.2%)	195 (23.4%)	0.601
	Manufacturer-origin report	Yes	441 (99.1%)	831 (99.6%)	−0.068
	United States report	Yes	398 (89.4%)	824 (98.8%)	−0.406
	Literature report	Yes	52 (11.7%)	2 (0.2%)	0.498
Procedural/use coding	Procedural/use composite (P1)	Yes	57 (12.8%)	171 (20.5%)	−0.208
Co-reported medication	Anticoagulant co-report	Yes	0 (0.0%)	8 (1.0%)	−0.139
	Antiplatelet co-report	Yes	13 (2.9%)	42 (5.0%)	−0.108
	PDE5 inhibitor co-report	Yes	22 (4.9%)	38 (4.6%)	0.018
Clinical phenotype	Surgery/repair phenotype (M1)	Yes	194 (43.6%)	30 (3.6%)	1.068
	Hematoma/hemorrhage phenotype (H1)	Yes	36 (8.1%)	653 (78.3%)	−2.009
	Functional impairment phenotype (S1)	Yes	7 (1.6%)	45 (5.4%)	−0.210
Outcome coding	Hospitalization code	Yes	71 (16.0%)	29 (3.5%)	0.431
Reporting process	Report period	2014–2017	140 (31.5%)	241 (28.9%)	0.056
	Report period	2018–2021	178 (40.0%)	475 (57.0%)	−0.344
	Report period	2022–2025	127 (28.5%)	118 (14.1%)	0.357

Values are n (%) unless otherwise stated. Age is reported as median [IQR], with the available n/N shown in each phenotype column. SMD, standardized mean difference. F1 and I1 are mutually exclusive report phenotypes. Any serious outcome coding is shown in Supplementary Table S2 because it was empirically invariant among F1 reports in this cohort and was not modeled as an independent discriminator. Percentages are report proportions, not incidence.

### 2.6 Secondary Firth adjustment and P1 component analyses

The main comparison included mutually exclusive F1 and I1 reports. Raw proportions and the crude odds ratio were presented first. Firth-penalized logistic regression then modeled F1 rather than I1 coding as the binary outcome, with P1 as the predictor of interest and fixed adjustment for report period, healthcare professional reporter, and manufacturer origin. This secondary adjustment quantified whether the descriptive contrast persisted after measured reporting-process differences; it was not intended to estimate a causal effect or risk. Firth’s adjusted-score method reduces first-order maximum-likelihood bias (Firth, 1993) and provides finite estimates with separation or sparse cells (Heinze and Schemper, 2002). Exponentiated coefficients are reported as adjusted reporting odds ratios (aRORs), with profile-likelihood 95% confidence intervals (CIs) and two-sided *p*-values.

Each P1 subdomain was examined separately and then in one model containing all four subdomains. These analyses were prespecified but exploratory, so nominal P values were retained. Leave-one-term-out analyses reconstructed P1 after removing each preferred term in turn. Calendar-specific models were adjusted for reporter and manufacturer origin, and a penalized likelihood-ratio test was performed to assess the P1-by-period interaction.

Robustness analyses used bleeding/contusion and pain/swelling I1 subsets, healthcare professional reports, and age-adjusted specifications. We also used all non-F1 reports as a broader comparator. A decontaminated report-richness model included literature source, US origin, log-transformed non-index preferred-term count, and log-transformed drug count. Covariates without variation in a subset were omitted and documented.

### 2.7 Severity contrasts and supportive hospitalization consistency

Two prespecified continuation-ratio contrasts represented the restricted ordering. The first compared any F1 with I1 without M1, and the second compared F1 with and without M1. Parallel overlapping contrasts compared any F1, F1 with M1, F1 with hospitalization, and F1 with both M1 and hospitalization against all I1 reports. Within-F1 models examined M1, hospitalization, and joint M1-plus-hospitalization coding, with P1 as the predictor and fixed core adjustment. The Holm correction was applied within the prespecified nested-outcome and within-F1 families.

Hospitalization provided an exploratory check of internal consistency across the three mutually exclusive rungs. The analysis included both a categorical model with L0 as the reference and a linear one-rung trend, with adjustment for the core reporting-process variables. It tested whether the restricted coding order tracked an independent serious-outcome code; it did not validate clinical progression or adjudicated injury severity. The excluded I1-with-M1 cell remained visible in the complete descriptive matrix and was not used to claim a global severity ordering.

### 2.8 Report-level therapy-start-to-event interval and selection model

The report-level therapy-start-to-event interval, retained as a time-to-onset (TTO) proxy for continuity with pharmacovigilance terminology, was calculated for target-drug sequences with exact eight-digit treatment-start and event dates. It was defined as the non-negative interval from the reported therapy start to the event date. When several qualifying CCH sequences were present, we calculated the interval for each sequence and retained the shortest non-negative value. FAERS does not reliably identify treatment-cycle boundaries, and a sequence start date need not be the date of the most recent injection before the event. The measure therefore cannot be interpreted as injection-to-fracture latency. The date-complete F1 subset was summarized using an empirical cumulative distribution. Prespecified, mutually exclusive windows were 0–7, 8–14, 15–28, and >28 days.

Because valid dates were incomplete, a separate Firth model among all F1 reports characterized entry into the date-complete subset. Predictors were M1, P1, report period, healthcare-professional reporter, manufacturer origin, age availability, log (1 + preferred-term count), and log (1 + drug count). This diagnostic model described, but did not correct, selection bias. The intervals were not treated as clinical time-to-event estimates.

### 2.9 Differential-under-recording tipping-point analysis

We used a one-way quantitative bias analysis with explicit assumptions and thresholds (Lash et al., 2014). It examined preferential under-recording of P1 in F1 while holding observed I1 coding fixed. For each integer k, currently P1-negative F1 reports were reclassified as P1-positive. Crude paths used two-sided Fisher exact tests, whereas adjusted paths preserved the measured covariate distribution. For the deterministic path, k reports were allocated proportionally across report-period, reporter, and manufacturer-origin strata. Largest-remainder apportionment preceded grouped mean-bias-reduced logistic regression.

Four thresholds were reported: a crude Fisher *p* ≥ 0.05, an adjusted 95% CI including 1, equal crude P1 proportions, and an adjusted point estimate ≥1. Allocation uncertainty was assessed by randomly reallocating k reports without replacement within the observed covariate structure. This analysis used k = 0–80, 1,000 repetitions per k, and seed 20260808. The tipping path measured fragility; it did not estimate how much under-recording occurred.

### 2.10 Clinical triangulation and same-indication comparator scan

We compared the present report phenotype qualitatively with four clinical sources. These were the current US prescribing information, pivotal trials, pooled clinical-development safety evidence, and a chart-reviewed Veterans Affairs cohort. A structured table recorded design, denominator, definite rupture or fracture, possible rupture or severe hematoma, timing, surgery or repair, and source-specific limitations. Because these designs were incompatible, patient denominators were not combined with spontaneous-report counts.

All latest-version strict Peyronie’s disease reports were also scanned for non-CCH primary-suspect therapies. Before analysis, a clinically coherent same-indication comparator had to include at least 50 reports and five F1 events. Simple covariate adjustment additionally required at least 10 F1 events in each exposure group. Heterogeneous residual therapies were not pooled merely to meet these thresholds.

### 2.11 Software, reproducibility, and ethics

The statistical analysis code is supplied as Supplementary File S4, and the complete frozen operational, phenotype, indication, product, and drug-name normalization dictionaries are supplied as Supplementary File S5. The dated protocol and SAP/amendment are provided as Supplementary Files S2, S3. Individual report identifiers, narratives, and raw report-level data are not redistributed.

The longitudinal terminology audit compared every frozen F1, I1, M1, and P1 string observed in the cohort with MedDRA version 29.0. The only substantive label transition was from the historical LLT “Inappropriate schedule of drug administration” to the current PT “Inappropriate schedule of product administration” (PT 10081572); both strings had been included in the frozen P1 dictionary. Frozen strings absent from version 29.0 contributed no reports. Thus, no observed defining concept was lost solely because of the terminology transition, although period-related coding practice can still change.

FAERS quarterly files are publicly accessible and contain de-identified reports. The study involved no intervention, reporter contact, or access to identifiable narratives; therefore, ethics committee approval and informed consent were not applicable.

## 3 Results

### 3.1 Cohort assembly and report characteristics

Scanning all quarterly archives identified 3,753 candidate reports with the strict Peyronie’s disease indication. Three FDA deletion-listed reports were excluded, leaving 3,750 latest-version strict reports. Collagenase *clostridium* histolyticum (CCH) was the sole primary suspect in 3,658 reports. The remaining 92 had another or no linked primary suspect. The deletion-list-aware active comparison comprised 1,279 mutually exclusive reports: 445 with the fracture-coded phenotype (F1) and 834 with the non-fracture local-injury phenotype (I1) (Figure 1). The other 2,379 CCH reports were not patients excluded for missing data; they remained outside the active contrast because none contained the frozen F1 term or an I1 qualifying term. Of these reports, 413 (17.4%), 1,179 (49.6%), and 787 (33.1%) were submitted in 2014–2017, 2018–2021, and 2022–2025, respectively; furthermore, 6 (0.3%) contained M1 coding and 65 (2.7%) contained hospitalization coding (Supplementary Table S2).

**FIGURE 1:**
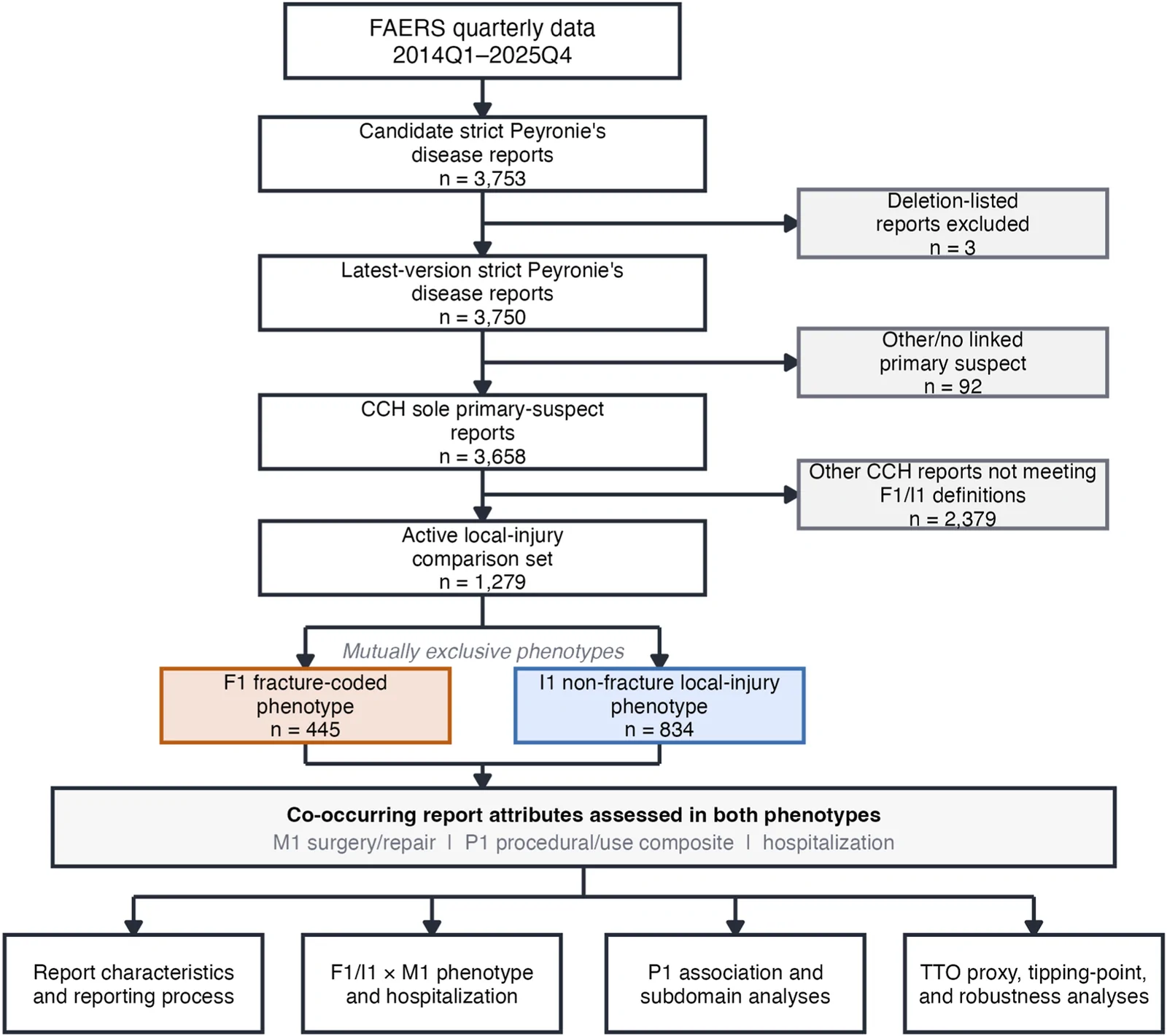
Study cohort assembly, injury-phenotype branching, and analysis framework. Flow diagram of cohort construction and prespecified analyses using FAERS quarterly data from 2014Q1 through 2025Q4. Among 3,753 candidate strict Peyronie’s disease reports, three deletion-listed reports were excluded, leaving 3,750 latest-version strict reports. Of these, 3,658 listed CCH as the sole primary suspect; 92 reports had another or no linked primary suspect. The active local-injury comparison set comprised 1,279 mutually exclusive reports with either the fracture-coded phenotype (F1; n = 445) or the non-fracture local-injury phenotype (I1; n = 834); the remaining 2,379 CCH reports met neither definition. Surgery/repair coding (M1), the heterogeneous procedural/use-reporting summary (P1), and hospitalization were assessed as co-occurring report attributes in both phenotypes. The four lower boxes map the prespecified result domains. Counts represent spontaneous reports, not patients or incidence.

Age was available for 213/445 (47.9%) F1 and 465/834 (55.8%) I1 reports. Among reports with age data, the median was 53 years (IQR 48–60) for F1 and 57 years (IQR 50–62) for I1. Healthcare professionals submitted 228/445 (51.2%) F1 and 195/834 (23.4%) I1 reports. Literature reports accounted for 52/445 (11.7%) F1 reports and 2/834 (0.2%) I1 reports. Almost all reports in both groups originated from manufacturers (441/445, 99.1% vs. 831/834, 99.6%). P1 coding was present in 57/445 (12.8%) F1 and 171/834 (20.5%) I1 reports (Table 1). Annual volume, reporter composition, age completeness, and therapy-start-to-event interval availability varied during the study period (Supplementary Figure S1; Supplementary Table S2).

### 3.2 Injury-by-management phenotype and hospitalization coding

Cross-classification of injury phenotype and surgery/repair coding (M1) produced four mutually exclusive cells. These comprised I1 without M1 (n = 804), I1 with M1 (n = 30), F1 without M1 (n = 251), and F1 with M1 (n = 194) (Figure 2A). M1 was recorded in 43.6% of F1 reports and 3.6% of I1 reports. Hospitalization appeared in 20/804 (2.5%), 9/30 (30.0%), 20/251 (8.0%), and 51/194 (26.3%) reports, respectively (Figure 2B).

**FIGURE 2:**
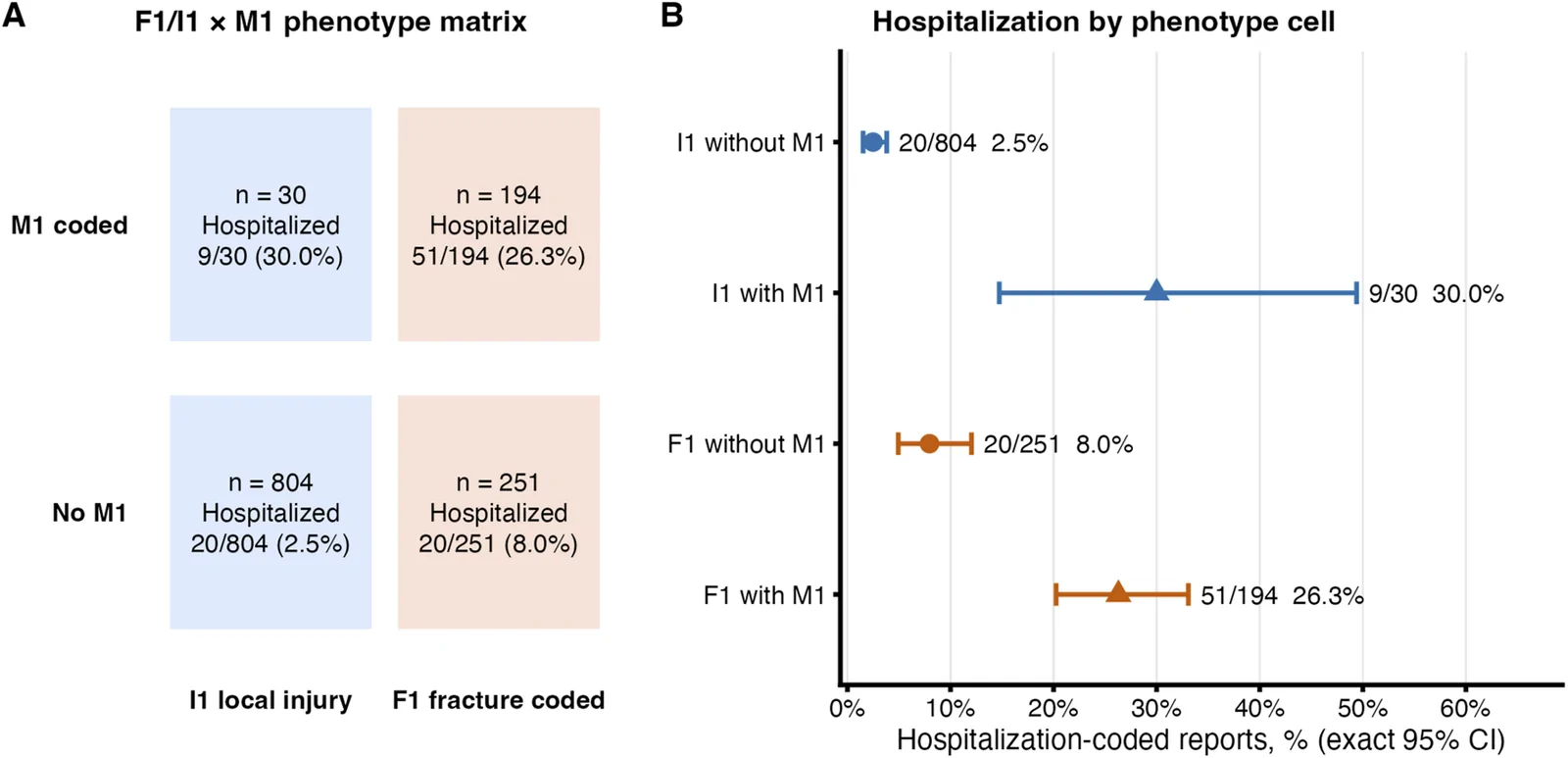
Complete injury-by-management phenotype and hospitalization coding. **(A)** Complete mutually exclusive F1/I1 × M1 phenotype matrix in the deletion-list-aware active comparison set. The four cells comprised I1 without M1 (n = 804), I1 with M1 (n = 30), F1 without M1 (n = 251), and F1 with M1 (n = 194). Hospitalization was coded in 20/804 (2.5%), 9/30 (30.0%), 20/251 (8.0%), and 51/194 (26.3%) reports, respectively. **(B)** Cell-specific hospitalization proportions with exact binomial 95% confidence intervals. Circles denote reports without M1 coding, and triangles denote reports with M1 coding; color distinguishes I1 from F1.

The 30 I1 reports with M1 remained in the complete phenotype description but were excluded from the prespecified restricted construct; their hospitalization proportion was 30.0%, illustrating why the construct is not a global clinical severity scale. The three included rungs were L0 (I1 without M1), L1 (F1 without M1), and L2 (F1 with M1). In the categorical Firth model, the adjusted reporting odds of hospitalization were 2.84-fold higher for L1 than L0 (95% CI: 1.43–5.62; *p* = 0.003) and 12.95-fold higher for L2 than L0 (95% CI: 7.51–23.16; *p* < 0.001). The corresponding linear estimate was 3.69 per rung (95% CI: 2.80–4.94; *p* < 0.001) (Table 2; Supplementary Table S5). These findings provide supportive internal consistency for the restricted coding hierarchy; they do not validate clinical severity or within-person progression.

**TABLE 2:** Observed P1 reporting and key secondary adjusted analyses.

Analysis role	Outcome contrast	Observed data	Analysis n	Crude odds ratio (95% exact CI)	Secondary adjusted reporting odds ratio (95% profile-likelihood CI)	*p*-value	Adjustment covariates
Primary descriptive and crude contrast	P1 reporting: F1 versus I1	F1: 57/445 (12.8%; 95% CI 9.8–16.3); I1: 171/834 (20.5%; 95% CI 17.8–23.4)	1,279	0.57 (0.40–0.80)	0.50 (0.35–0.71)	Crude <0.001; adjusted <0.001	Report period + healthcare-professional reporter + manufacturer origin
Supportive categorical hospitalization consistency	L1 (F1 without M1) versus L0 (I1 without M1)	L1: 20/251 (8.0%); L0: 20/804 (2.5%)	1,249	3.39 (1.70–6.76)	2.84 (1.43–5.62)	0.003	Report period + healthcare-professional reporter + manufacturer origin
Supportive categorical hospitalization consistency	L2 (F1 with M1) versus L0 (I1 without M1)	L2: 51/194 (26.3%); L0: 20/804 (2.5%)	1,249	13.92 (7.88–25.44)	12.95 (7.51–23.16)	<0.001	Report period + healthcare-professional reporter + manufacturer origin
Supportive linear trend	Hospitalization per one-rung increase from L0 to L2	L0: 20/804 (2.5%); L1: 20/251 (8.0%); L2: 51/194 (26.3%)	1,249	Not applicable to the ordinal trend	3.69 (2.80–4.94)	<0.001	Report period + healthcare-professional reporter + manufacturer origin

All rows use the deletion-list-aware cohort. Raw proportions with exact binomial 95% confidence intervals and crude Fisher odds ratios are foregrounded. Adjusted estimates are secondary Firth models controlling for report period, healthcare professional reporter, and manufacturer origin. Categorical ladder rows provide supportive internal consistency with hospitalization coding and do not validate clinical severity. Estimates are not incidence, risk ratios, causal effects, or protection. P1, procedural/use-reporting summary; M1, surgery/repair coding.

### 3.3 Observed P1 reporting and secondary adjusted analysis

P1 was observed in 57/445 F1 reports (12.8%, exact 95% CI: 9.8%–16.3%) and 171/834 I1 reports (20.5%, 17.8%–23.4%). The crude reporting odds ratio was 0.57 (exact 95% CI: 0.40–0.80; Fisher *p* < 0.001) (Figure 3A; Table 2). After secondary adjustment for reporting period, healthcare-professional reporter, and manufacturer origin, the aROR was 0.50 (95% CI: 0.35–0.71; *p* < 0.001) (Figure 3B; Table 2). Adjustment therefore strengthened the inverse association modestly but did not change its direction or qualitative conclusion. Its specific added value was control of measured period and reporter-type imbalance, not estimation of a causal effect or risk. Healthcare professional reporting was associated with F1 rather than I1 coding (aROR 3.24, 95% CI: 2.50–4.21), and the corresponding aROR was 2.22 (95% CI: 1.58–3.15) for 2022–2025 relative to 2014–2017 (Supplementary Table S3).

**FIGURE 3:**
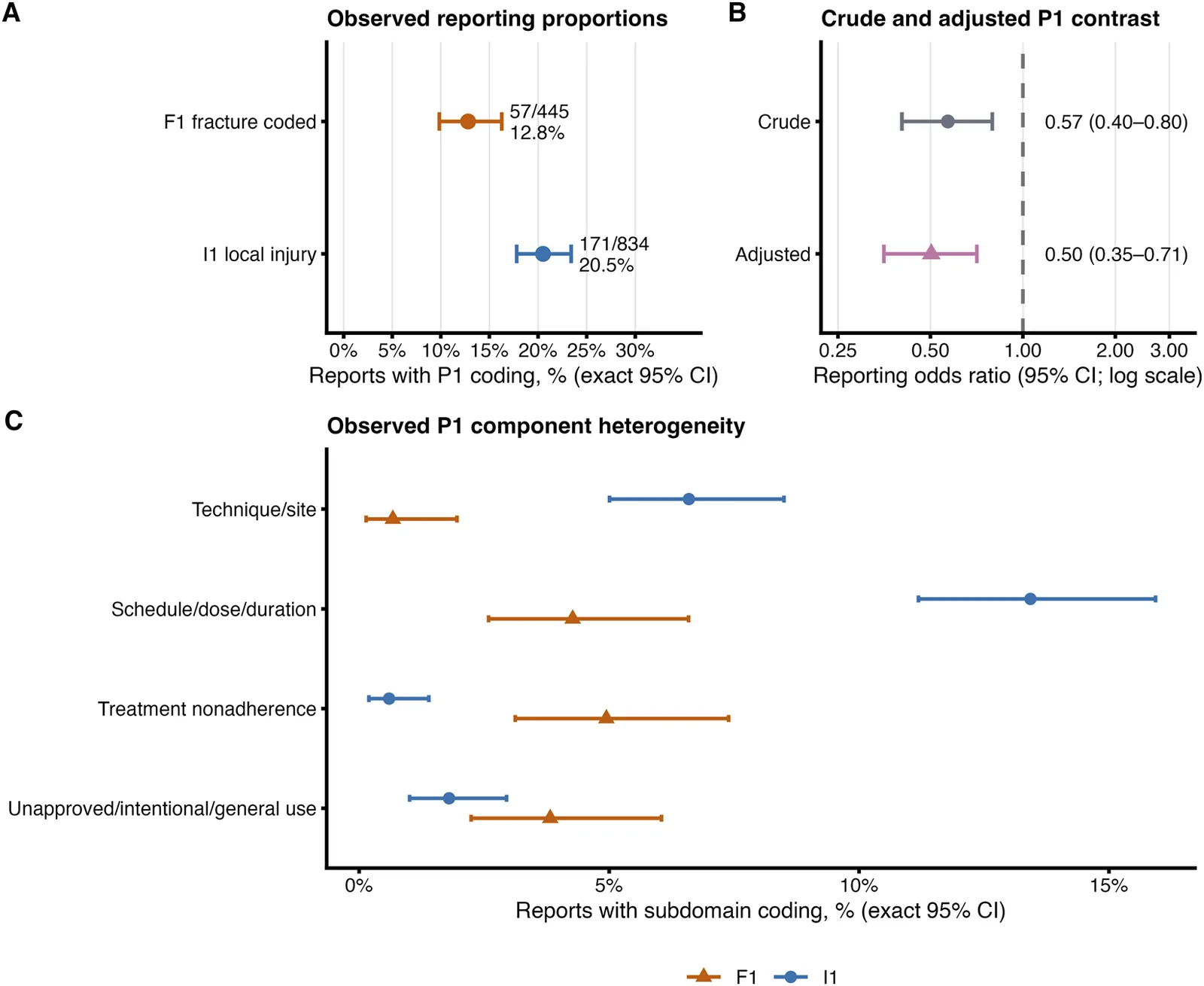
Observed P1 reporting, crude and adjusted contrasts, and component heterogeneity. **(A)** Observed P1 proportions in F1 and I1 reports with exact binomial 95% confidence intervals. **(B)** The crude reporting odds ratio from a two-sided Fisher exact test and the secondary reporting odds ratio adjusted for report period, healthcare-professional reporter, and manufacturer origin using Firth logistic regression. The adjustment controls measured reporting-process imbalance; it does not estimate a causal effect or risk. **(C)** Observed P1 subdomain proportions with exact binomial 95% confidence intervals. The direction differed across technique/site, schedule/dose/duration, treatment nonadherence, and unapproved/intentional/general-use coding, showing that P1 is a heterogeneous reporting-process summary rather than a unitary clinical construct.

The observed subdomain proportions were directionally discordant (Figure 3C). Technique/site coding occurred in 3/445 (0.7%) F1 and 55/834 (6.6%) I1 reports, and schedule/dose/duration coding occurred in 19/445 (4.3%) and 112/834 (13.4%), respectively. Conversely, treatment-nonadherence coding was more frequent in F1 (22/445, 4.9%) than in I1 reports (5/834, 0.6%); unapproved/intentional/general-use coding occurred in 17/445 (3.8%) and 15/834 (1.8%). Thus, the aggregate P1 contrast does not represent a single coherent clinical direction. Adjusted subdomain, leave-one-term-out, and period-specific models are reported as secondary analyses in Supplementary Figure S2 and Supplementary Table S4. In the report-richness model that additionally included literature source and US origin, the aggregate estimate remained inverse (aROR 0.41, 95% CI: 0.27–0.61) (Supplementary Table S3).

Secondary severity and nested-outcome models are reported in Supplementary Figure S3 and Supplementary Table S5. These models did not replace the observed four-cell phenotype matrix or the raw P1 proportions as the principal results.

### 3.4 Therapy-start-to-event interval and selection into the date-complete subset

A non-negative report-level therapy-start-to-event interval, retained as a TTO proxy, was available for 91/445 (20.4%) F1 reports. Date completeness was associated with age availability (aROR 25.28, 95% CI: 10.78–69.17) and healthcare professional reporting (aROR 2.56, 95% CI: 1.37–4.90). Preferred-term count was also associated with completeness (aROR 0.24 per log unit, 95% CI: 0.09–0.60). The corresponding aROR for drug count was 2.13 per log unit (95% CI: 1.26–3.66). The aROR for P1 coding was 0.25 (95% CI: 0.07–0.72) (Figure 4C; Supplementary Table S6). Selection into the date-complete subset was therefore non-random across measured report characteristics.

**FIGURE 4:**
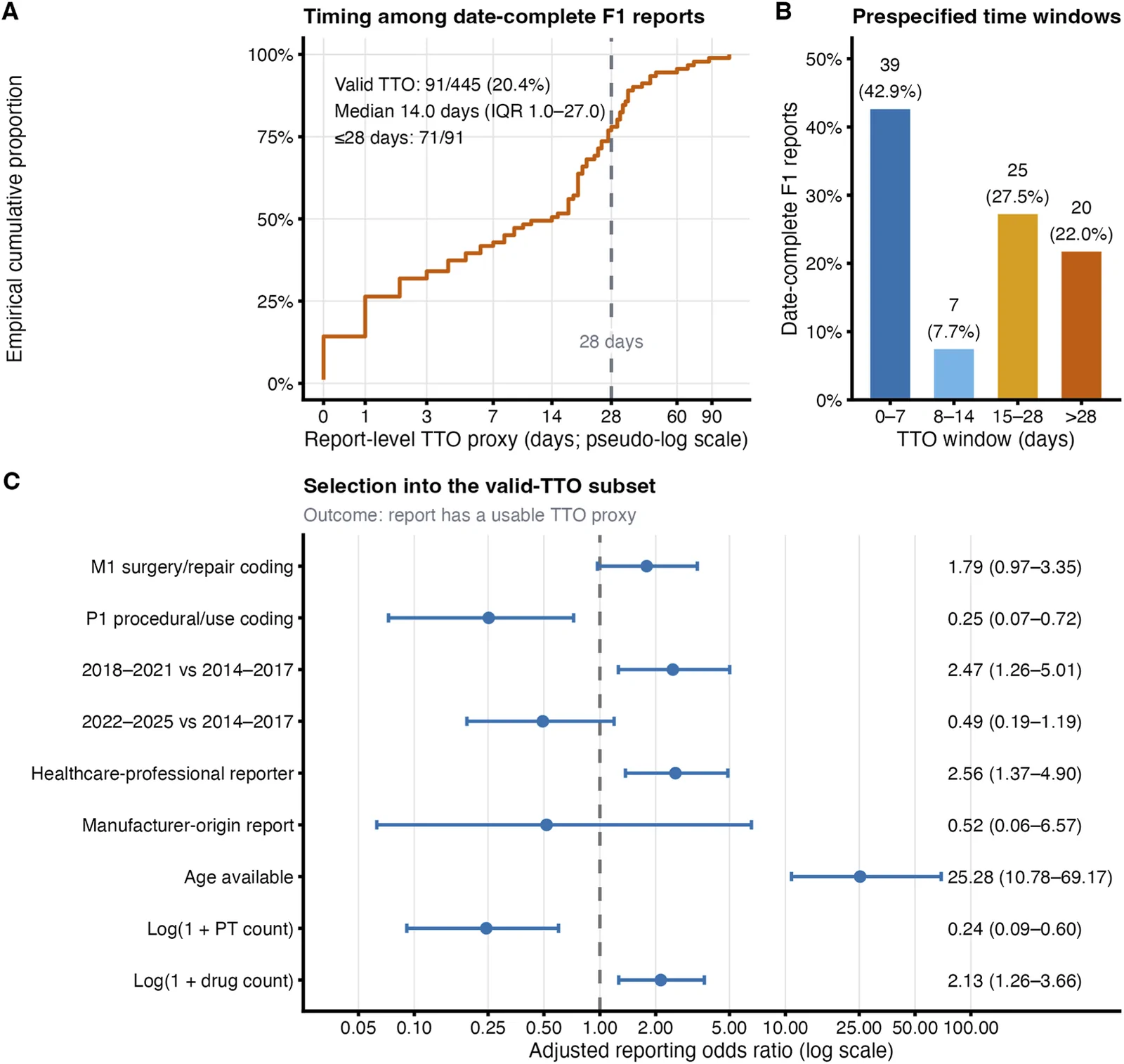
Therapy-start-to-event interval among F1 reports and selection into the date-complete subset. **(A)** Empirical cumulative distribution of the report-level therapy-start-to-event interval, retained as a time-to-onset (TTO) proxy, among 91/445 (20.4%) F1 reports with usable dates. The median was 14 days (IQR 1–27), and 71/91 intervals were ≤28 days. The horizontal axis uses a pseudo-log transformation to retain day 0. **(B)** Prespecified intervals: 0–7 days, 39/91 (42.9%); 8–14 days, 7/91 (7.7%); 15–28 days, 25/91 (27.5%); and >28 days, 20/91 (22.0%). **(C)** Firth penalized logistic regression of selection into the date-complete subset among all F1 reports. FAERS does not reliably identify treatment-cycle boundaries, and the reported start date need not be the latest injection; panels A and B, therefore, do not estimate injection-to-fracture or patient-level latency.

Among the 91 date-complete F1 reports, the median therapy-start-to-event interval was 14 days (IQR 1–27). An interval of 28 days or less was recorded in 71/91 (78.0%) reports (Figure 4A). The 0–7-day window contained 39/91 (42.9%) reports, and the 8–14-day window contained 7/91 (7.7%). The 15–28-day and >28-day windows contained 25/91 (27.5%) and 20/91 (22.0%) reports, respectively (Figure 4B). The median was 5 days (IQR 1–23) among 37 F1 reports without M1 and 17 days (IQR 3.2–29.5) among 54 F1 reports with M1. Because FAERS does not reliably identify treatment-cycle boundaries and the reported start date need not represent the latest injection, these intervals cannot be interpreted as injection-to-fracture latency or as an unbiased clinical latency distribution.

### 3.5 Differential P1 under-recording boundary and robustness

The one-way tipping-point analysis reclassified observed P1-negative F1 reports as P1-positive while preserving the measured covariate distribution. Reclassifying 14 reports (3.1% of F1) made the crude Fisher test non-significant (*p* = 0.051). At 20 reports (4.5%), the balanced adjusted 95% CI first included 1 (aROR 0.74, 95% CI: 0.53–1.01). Reclassification of 35 reports (7.9%) equalized the crude P1 proportions. At 41 reports (9.2%), the adjusted point estimate reached 1.01 (95% CI: 0.75–1.36) (Figure 5A; Supplementary Table S7). Random-allocation simulations yielded a concordant boundary. At k = 20, 73.7% of fitted 95% CIs included 1, and this proportion reached 100% at k = 35. At k = 41, the median OR was 1.01% and 70.7% of point estimates were at or above 1 (Supplementary Table S7).

**FIGURE 5:**
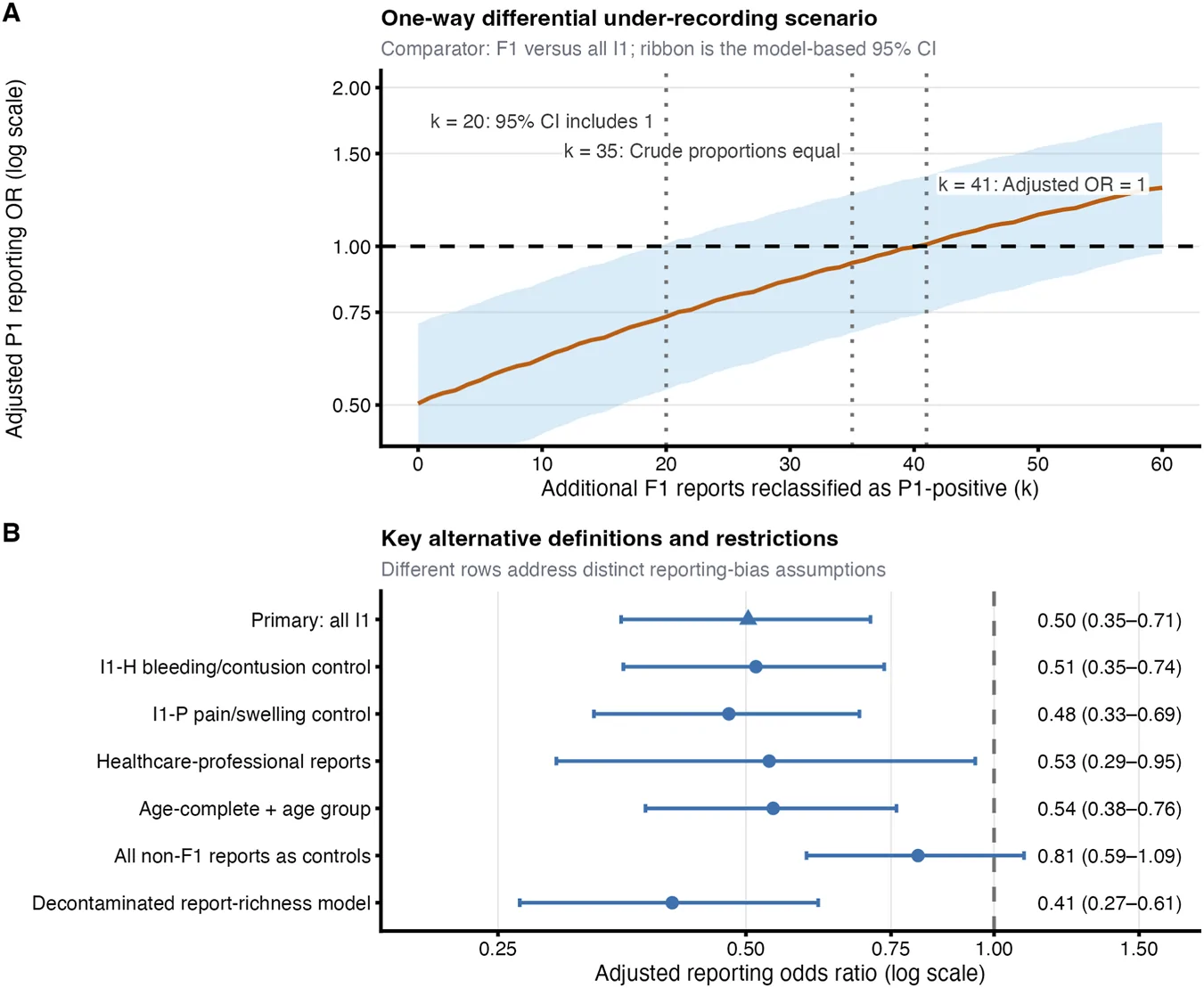
Differential P1 under-recording boundary and key robustness analyses. **(A)** One-way tipping-point analysis in the deletion-list-aware active comparison set, comparing F1 with all I1 reports. The scenario sequentially reclassified k observed P1-negative F1 reports as P1-positive while retaining the observed covariates. The model-based 95% CI first included 1 at k = 20, the crude P1 proportions were equal at k = 35, and the adjusted reporting OR reached 1 at k = 41. Random-allocation uncertainty is reported in Supplementary Table S7. **(B)** P1 aRORs under alternative local-injury comparators, reporter restrictions, missing-category and complete-case age models, an all-non-F1 comparator, and a decontaminated report-richness model. Each row addresses a distinct reporting-process assumption and should not be interpreted as an independent confirmatory analysis. Points show Firth aRORs, and horizontal lines show profile-likelihood 95% CIs.

The estimate was similar to bleeding/contusion comparators (aROR 0.51, 95% CI: 0.35–0.74). It was also similar to pain/swelling comparators (aROR 0.48, 95% CI: 0.33–0.69). The aROR was 0.53 (95% CI: 0.29–0.95) among healthcare-professional reports. The missing-category age model remained inverse (aROR 0.54, 95% CI: 0.38–0.76; *p* < 0.001). The complete-case age model was attenuated, and its CI included 1 (n = 678; aROR 0.72, 95% CI: 0.46–1.11; *p* = 0.140). Using all non-F1 reports as controls moved the estimate toward the null (aROR 0.81, 95% CI: 0.59–1.09). A decontaminated report-richness model that additionally controlled for literature source, US origin, non-index preferred-term count, and drug count produced an aROR of 0.41 (95% CI: 0.27–0.61), supporting robustness to measured report-source and report-richness differences (Figure 5B; Supplementary Table S3). Deletion-list exclusion changed F1 from 446 to 445 and F1 with M1 from 195 to 194; these deletion-aware counts are used consistently throughout the tables and figures. Neither the primary estimate nor the hospitalization gradient changed materially (Supplementary Table S7). Independent bias-reduced fits agreed with the Firth estimates to three decimals, and every prespecified model converged (Supplementary Table S3).

### 3.6 Clinical evidence triangulation and same-indication comparator feasibility

Regulatory and clinical sources described timing and management dimensions that were also visible in the report phenotype, but their definitions, time origins, and denominators were not comparable (Table 3). The US prescribing information reports 5 definite ruptures among 1,044 treated participants. It also reports 9 cases in which rupture could not be excluded and 39 severe penile hematomas (U.S. Food and Drug Administration, 2023). All three ruptures in the pivotal trials underwent repair (Gelbard et al., 2013). The pooled six-study safety analysis recorded four treatment-related serious ruptures and five serious penile hematomas under publication-specific definitions (Carson et al., 2015). In the Veterans Affairs cohort, 11/1,541 CCH-treated veterans had chart-confirmed rupture. Median time to rupture was 8 days; six underwent repair, and five were managed conservatively (Zucker et al., 2023). These sources provide clinical context but do not validate the FAERS coding hierarchy, establish direct temporal concordance, or convert report proportions into incidence estimates.

**TABLE 3:** Clinical evidence triangulation and design-specific comparison boundaries.

Evidence source	Design and denominator	Definite fracture or rupture	Possible rupture or severe hematoma	Timing	Surgery or repair	Comparison boundary
(U.S. Food and Drug Administration, 2023) FDA XIAFLEX prescribing information (2023)	Controlled and uncontrolled clinical studies; 1,044 treated patients and 7,466 injections	5/1,044 (0.5%) corporal rupture adverse reactions	9/1,044 (0.9%) with symptom combinations for which rupture could not be excluded; 39/1,044 (3.7%) severe penile hematoma	Individual rupture onset not reported	Total surgery count for the five definite ruptures not reported; all nine possible ruptures were managed without surgery	Known boxed-warning/REMS risk; clinical incidence is not comparable with spontaneous-report proportions
(Gelbard et al., 2013) IMPRESS I and II pivotal trials	Two randomized double-blind placebo-controlled phase 3 trials; 832 treated/evaluable participants (CCH 551; placebo 281)	3/551 CCH and 0/281 placebo corporal ruptures	33/551 (6.0%) severe penile/injection-site hematoma in CCH; 0/281 placebo	Rupture onset day not reported in the primary abstract	All three ruptures underwent surgical repair	Prospective active collection in a selected stable-disease population; overlaps the FDA development program
(Carson et al., 2015) Six-study pooled clinical safety analysis	Three randomized and three open-label studies; 1,044 patients received at least one CCH dose	Four treatment-related serious corporal ruptures in the publication’s narrower SAE category	Five treatment-related serious penile hematomas	Not reported in the abstract	Not reported in the abstract	Overlaps the pivotal/open-label studies and is not an independent validation cohort; regulatory and publication event scopes differ
(Zucker et al., 2023) Veterans Affairs cohort	Retrospective code screen plus individual chart review; 1,541 CCH-treated veterans among 17,647 with Peyronie’s disease	11/1,541 (0.7%) corporal ruptures	Not studied	Median 8 days; most within 2 weeks and associated with intercourse or spontaneous erection	Six surgical repairs; five conservative management	Provides real-world timing and management context; time origin, population, ascertainment, and comparator structure differ
Present study	3,658 deletion-list-aware CCH primary-suspect Peyronie’s disease reports; active local-injury comparison set n = 1,279; no exposed-patient denominator	445 F1 fracture-coded reports	Not adjudicated; 834 mutually exclusive non-F1 local-injury reports formed the active comparator; report phenotype rather than an incidence endpoint	Usable therapy-start-to-event interval in 91/445 (20.4%); median 14 days (IQR 1–27); 71/91 within 28 days; not injection-to-fracture latency	194/445 had M1 surgery/repair coding; 71/445 had hospitalization coding; 51 had both	Internal report-phenotype and severity structure only; report proportions are not incidence

Clinical-study and spontaneous-report denominators are not directly comparable. Report proportions are not incidence estimates. Parenthetical numbers are Vancouver references in the manuscript (U.S. Food and Drug Administration, 2023): FDA XIAFLEX prescribing information (Gelbard et al., 2013); IMPRESS I and II (Carson et al., 2015); pooled clinical safety analysis (Zucker et al., 2023); and Veterans Affairs cohort. CCH, collagenase *clostridium* histolyticum; TTO, time-to-onset proxy.

The all-Peyronie’s-disease scan identified 54 non-CCH primary-suspect reports across heterogeneous therapies, with no F1 event. The largest alternative-therapy cell contained only 14 reports, and no coherent comparator reached 50 reports with five F1 events. A same-indication treatment-comparison model was, therefore, not fitted (Supplementary Table S8).

## 4 Discussion

This indication-restricted analysis treated fracture coding after CCH as a known serious risk rather than a newly detected signal. The reports revealed a clinically recognizable but heterogeneous injury phenotype. Surgery/repair and hospitalization were often recorded with F1, and hospitalization increased across the restricted report-level severity ordering. The primary descriptive comparison showed P1 in 12.8% of F1 reports and 20.5% of I1 reports (crude OR 0.57, 95% CI: 0.40–0.80). Secondary adjustment for reporting period, healthcare-professional reporter, and manufacturer origin modestly strengthened the estimate (aROR 0.50, 95% CI: 0.35–0.71) without changing its direction. P1 component domains nevertheless differed markedly in direction, and the aggregate association crossed conventional statistical thresholds after modest one-way reclassification. Its magnitude therefore depended partly on which contextual details reporters recorded.

Fracture and management codes were related at the report level but were not interchangeable. Nearly half of F1 reports also contained an M1 term, yet many did not. Conversely, 30 I1 reports contained M1, and hospitalization was frequent in that small cell. This pattern could reflect differences in presentation, diagnostic certainty, management, or coding practice. Late-onset rupture has also been documented after CCH (Wainstein et al., 2025). CCH outcomes can vary by sexual population (Miller et al., 2026), curvature location (Larson et al., 2024), and disease phase (Kuchakulla et al., 2025). Real-world series document incomplete treatment adherence (Lumbiganon et al., 2025) and substantial discontinuation, even in high-volume care (Lehner et al., 2023). The L0–L2 construct should therefore be interpreted as a restricted ordering of reports with supportive internal consistency from hospitalization coding. The 30% hospitalization proportion in the excluded I1-with-M1 cell directly precludes treating it as a global severity scale. It is not a biological sequence, a validated clinical grade, or a clinical triage rule.

Surgery/repair coding also sits within a broader treatment pathway. Some veterans undergo surgery after CCH (Zucker et al., 2022), while long-term satisfaction may coexist with persistent or remembered adverse effects (Song et al., 2024). The available evidence for combination strategies remains heterogeneous (Guo et al., 2024). A randomized comparison of CCH and surgery found distinct efficacy, adverse-event, recovery, and preference profiles (Green et al., 2023). These studies make the M1 coding clinically relevant, but they cannot verify that a spontaneous-report term represents a completed operation. Nor can M1 be used as a direct injury grade.

The P1 result is best understood as a high-sensitivity summary of reporting-process detail, not as a unitary clinical construct. Technique/site and schedule/dose/duration terms were much less frequent in F1 than in I1, whereas treatment-nonadherence terms were more frequent. A single composite estimate therefore conceals important differences among its components. More importantly, a lower P1 recording with F1 cannot indicate procedural protection. A salient injury may dominate a report and displace less prominent contextual details. Reporters may also differ in their access to procedure information. Regulatory attention can further stimulate reporting of a named serious event, and notoriety has altered both report volume and signal strength in FAERS (Neha et al., 2019). Claims and practice data show that CCH treatment and downstream care occur in selected populations (Trost et al., 2021). Surgery after unsuccessful CCH also represents a selected clinical subset (Cocci et al., 2021). Acute-phase cohorts (Hu et al., 2022), response-prediction studies (Flores et al., 2022), pooled cycle-response analyses (Ziegelmann et al., 2023), and prospective outcome series (Cahill and Trost, 2025) mainly address effectiveness and treatment selection. They cannot determine why a procedural term is absent from a spontaneous report.

The tipping-point analysis places a quantitative boundary around this uncertainty. Reclassifying 20 P1-negative F1 reports, or 4.5% of the F1 set, brought the balanced adjusted confidence interval across 1. Thirty-five reports equalized the crude proportions, and 41 moved the adjusted point estimate to approximately 1. These values do not estimate the number of misclassified reports. They show how little differential recording would be needed to remove the inverse association. The analysis was intentionally one-sided because the main concern was under-recording of contextual procedure or use terms when a severe fracture term was present. Other forms of misclassification remain possible.

Clinical evidence was used for context rather than statistical replication. The US label and pooled development program already identify rupture and severe penile hematoma as serious adverse reactions (Carson et al., 2015; U.S. Food and Drug Administration, 2023). All ruptures reported in the pivotal trials underwent repair (Gelbard et al., 2013). The Veterans Affairs cohort reported a median of 8 days to rupture and included both surgical and conservative management (Zucker et al., 2023). In FAERS, the median report-level therapy-start-to-event interval was 14 days among date-complete F1 reports, but direct numerical comparison is inappropriate: repeated treatment cycles are common, the FAERS start date may not be the most recent injection, and usable dates were available for only one-fifth of F1 reports. Date completeness also varied with age availability, reporter type, report richness, and P1 coding. The timing results therefore provide descriptive report-level context, not injection-to-fracture latency or an unbiased patient-level latency curve.

The crude and adjusted P1 estimates answered related but distinct questions. The crude OR summarized the observed contrast between F1 and I1 reports and is therefore the primary result. The secondary Firth model controlled for measured imbalance in calendar period, healthcare professional reporting, and sparse manufacturer origin while reducing small-sample coefficient bias. The change from 0.57 to 0.50 was modest and did not materially alter the conclusion. Adjustment was not intended to recover a causal treatment effect, estimate risk, or make the two reporting phenotypes exchangeable. Literature sources and U.S. origin were reserved for sensitivity analysis because they were sparse or strongly imbalanced and could destabilize the core model; the expanded decontaminated model nevertheless remained inverse after adding these variables and report-richness measures.

Several design features improved interpretability compared with an unrestricted spontaneous-report screen. Product, indication, primary-suspect role, report version, and deletion-list status were fixed before phenotype analysis. The comparator was a mutually exclusive local-injury phenotype within the same indication. This choice reduced some disease- and treatment-context differences, although comparator selection remains a major determinant of pharmacovigilance estimates (Gravel and Douros, 2023). An active report comparator cannot make a disproportionality estimate causal (Scosyrev et al., 2025). Current guidance instead supports spontaneous-report data for signal detection and characterization, not incidence estimation or stand-alone causal inference (Cutroneo et al., 2024). We also retained the complete injury-by-management matrix before imposing the restricted severity ordering. Sparse cells were handled with Firth regression, entry into the date-complete subset was modeled, and P1 was examined by component and period. The tipping analysis quantified a specific recording-bias threshold rather than treating under-recording as a generic limitation.

The study nevertheless retains the limitations of FAERS. There is no exposed-patient denominator, and diagnoses, procedures, and clinical chronology are not verified. Reaction terms, outcome codes, dates, and management also lack consistent event-level linkage. Manufacturer-origin reports dominated both groups. Several covariates were sparse, age was often missing, and date completeness was low and non-random. The complete-case age estimate was attenuated and imprecise, whereas the missing-category model remained inverse; neither approach resolves potentially informative age missingness. Latest-version handling cannot exclude every residual duplicate. Duplicate reports have recognizable features in FAERS (Janiczak et al., 2025), and network-based grouping can alter how related submissions are assembled (Kreimeyer et al., 2025). Duplication also affects pharmacovigilance databases more broadly (Kiguba et al., 2024). Coding competition, severity-driven documentation, stimulated reporting, litigation, publicity, and unmeasured clinical differences could all shape the observed phenotype. MedDRA labels and coding practice can also change over time. Our version 29.0 audit found that the observed historical label “Inappropriate schedule of drug administration” mapped to the current preferred term “Inappropriate schedule of product administration” (PT 10081572), and both labels were included in the frozen P1 dictionary; no observed defining concept was lost in this transition. This audit reduces, but cannot eliminate, longitudinal terminology drift or changes in coding behavior. Methodological reviews describe how these processes distort disproportionality estimates (Fusaroli et al., 2025). Adverse drug reactions are also widely underreported, with variation across events and reporters (García-Abeijon et al., 2023). Finally, no coherent same-indication alternative met the prespecified report and event thresholds. Comparative treatment safety could not be estimated and was not forced from sparse data.

The principal contribution is a more precise reporting phenotype. FAERS represents this known serious injury through a heterogeneous combination of fracture, management, hospitalization, and timing codes. The inverse P1 association was too sensitive to differential recording to support a protective interpretation. For pharmacovigilance, the results favor preserving management detail, examining missing contextual codes, and testing reporting-bias boundaries before proposing mechanisms. For clinical care, they support counseling and prompt assessment of suspected serious penile injury, while diagnosis and management remain patient-level decisions.

## Data Availability

The original contributions presented in the study are included in the article/Supplementary Material, further inquiries can be directed to the corresponding authors.
