# Supplementary material for: The reporting phenotype of penile fracture after collagenase clostridium histolyticum for Peyronie’s disease: an indication-restricted FAERS study
Source: Front Pharmacol. 2026 Oct 14;17:1965417. doi: 10.3389/fphar.2026.1965417 (PMC13713637; doi:10.3389/fphar.2026.1965417)
Supplement: Supplementary file 1 [file Supplementaryfile1.docx]

**Supplementary Material**

# Contents

**Supplementary Figures**

Figure S1. Annual reporting process and data completeness	2

Figure S2. Detailed analyses of P1 composition and robustness	3

Figure S3. Restricted severity ladder and nested supportive outcomes	4

**Supplementary Tables**

Table S1. Operational definitions, analysis sets, variables, and time definitions	5

Table S2. Expanded report characteristics, completeness, annual reporting process, and reports outside the active comparison	7

Table S3. Complete primary, sensitivity, and diagnostic model results	10

Table S4. P1 subdomain frequencies and robustness analyses	11

Table S5. Restricted coding ladder, hospitalization, and nested-outcome analyses	13

Table S6. Therapy-start-to-event interval distribution and selection analysis	15

Table S7. P1 tipping-point and deletion-list sensitivity	17

Table S8. Same-indication comparator feasibility and data reconciliation	20

# Supplementary Figures

## Supplementary Figure S1. Annual reporting process and data completeness

(A) Annual active-comparison report counts, stacked by F1 and I1 phenotype. (B) Annual proportions of healthcare-professional and literature reports. (C) Annual completeness of age in all active-comparison reports and of the therapy-start-to-event interval among F1 reports. Annual fluctuations describe the reporting process and database completeness rather than changes in incidence.

## Supplementary Figure S2. Detailed analyses of P1 composition and robustness

(A) Secondary adjusted models for the four P1 subdomains, shown separately with core adjustment and mutually adjusted for the other subdomains. (B) Leave-one-preferred-term-out analyses in which P1 was reconstructed after removing one component term at a time; estimates are from Firth models adjusted for reporting period, healthcare-professional reporter, and manufacturer origin. (C) Calendar-period-specific P1 estimates adjusted for reporter and manufacturer source within each period. Points show aRORs and horizontal lines show profile-likelihood 95% CIs. Observed subdomain frequencies are foregrounded in Figure 3C and Table S4.

## Supplementary Figure S3. Restricted severity ladder and nested supportive outcomes

(A) Hospitalization proportions across the restricted compatible report-level ladder: L0, I1 without M1 (n = 804); L1, F1 without M1 (n = 251); and L2, F1 with M1 (n = 194). (B) P1 proportions across the same three mutually exclusive levels. Exact binomial 95% CIs are shown in panels A and B. The 30 I1-with-M1 reports were retained in the complete phenotype analysis (Figure 2) but excluded from this restricted ladder because they were incompatible with its ordering assumption. (C) P1 aRORs for nested and overlapping supportive outcome definitions. These rows are not mutually exclusive severity levels and are presented as supportive sensitivity analyses only.

**Table S1. Operational definitions, analysis sets, variables, and time definitions**

| **Category** | **Item** | **Definition** | **Analytic role** |
| --- | --- | --- | --- |
| Phenotype | F1 fracture-coded phenotype | Report contains the MedDRA preferred term Fracture of penis. | Case phenotype in the active F1-versus-I1 comparison. |
| Phenotype | I1 non-fracture local-injury phenotype | Non-F1 report containing at least one prespecified local-injury term: penile haematoma; penile haemorrhage; penile contusion; penile swelling; penile pain; genital haemorrhage; penile oedema; genital contusion; genital swelling; genital haematoma; genital pain; penis injury; penile injury; penile ecchymosis; penile laceration. | Active local-injury comparator; mutually exclusive with F1. |
| Co-occurring attribute | M1 surgery/repair coding | At least one surgery/repair term: corpora cavernosa surgery; penile operation; penile surgery. | Management/severity attribute; not mutually exclusive with F1 or I1. |
| Co-occurring attribute | P1 procedural/use summary | At least one heterogeneous procedural, use, schedule, or adherence term: wrong technique in product usage process; product administration error; inappropriate schedule of product administration; inappropriate schedule of drug administration; treatment noncompliance; off label use; product use in unapproved indication; intentional product use issue; incorrect dose administered; incorrect product administration duration; product administered at inappropriate site; product use issue. | High-sensitivity reporting-process summary and tipping-point target; not a unitary clinical construct. |
| P1 subdomain | Technique/site | wrong technique in product usage process; product administration error; product administered at inappropriate site | Prespecified P1 subdomain. |
| P1 subdomain | Schedule/dose/duration | inappropriate schedule of product administration; inappropriate schedule of drug administration; incorrect dose administered; incorrect product administration duration | Prespecified P1 subdomain. |
| P1 subdomain | Treatment nonadherence | treatment noncompliance | Prespecified P1 subdomain. |
| P1 subdomain | Unapproved/intentional/general use | off label use; product use in unapproved indication; intentional product use issue; product use issue | Prespecified P1 subdomain. |
| Outcome | Hospitalization | FAERS outcome code indicating hospitalization or prolonged hospitalization. | Supportive internal-consistency check; not a mutually exclusive rung or validated severity outcome. |
| Ordered construct | Restricted coding ladder | L0: I1 without M1; L1: F1 without M1; L2: F1 with M1. I1 with M1 is excluded and retained descriptively. | Restricted report-coding construct assessed for internal consistency with hospitalization; not a global clinical severity scale. |
| Time | Report-level therapy-start-to-event interval | Shortest non-negative difference between an available target-therapy start date and event date when more than one qualifying sequence was present. | Descriptive report-level proxy; treatment-cycle boundaries and the most recent injection date are not reliably identified. |
| Time | Date-complete subset | F1 reports with a usable non-negative therapy-start-to-event interval. | Interval distribution and selection model; not injection-to-fracture latency. |
| Period | Report period | 2014–2017, 2018–2021, or 2022–2025 based on report year. | Core adjustment and calendar-period stratification. |
| Analysis set | Primary active comparison set | Deletion-list-aware reports meeting either F1 or I1, n=1,279 (F1=445; I1=834). | Primary case–case models and descriptive comparisons. |
| Analysis set | Strict ladder set | Primary active comparison set after excluding 30 I1+M1 reports, n=1,249. | Strict severity-ladder transitions and ordered hospitalization model. |
| Feasibility rule | Same-indication comparator | A coherent alternative therapy must have at least 50 reports and at least five F1 events before comparative modelling is considered. | Prevents forced sparse comparator estimates. |
| Data source | Analysis period | FAERS quarterly files from 2014Q1 through 2025Q4. | Defines the temporal coverage of all analyses. |
| Exposure rule | Target therapy role | Eligibility required CCH to be linked as a primary suspect drug in a strict Peyronie's disease report. All primary-suspect sequences were enumerated; none of the 3,658 eligible reports contained a co-primary-suspect drug, so sole-primary-suspect status was an observed cohort property rather than an additional exclusion. | Restricts the study to target-therapy primary-suspect reports and distinguishes eligibility from an observed property. |
| Report versioning | Latest report version and deletion handling | For each report, the latest available version was retained and reports listed in FDA deleted-case files were excluded. | Reduces duplicate-version and deleted-report contamination. |
| Terminology | MedDRA preferred-term coding | Preferred-term labels as present in the quarterly FAERS files were mapped using the prespecified frozen dictionary; a single harmonized MedDRA release was not imposed. | States the coding convention and its reproducibility boundary. |
| Terminology | Longitudinal MedDRA audit | Observed frozen strings were checked against MedDRA v29.0. The historical label 'Inappropriate schedule of drug administration' mapped to current PT 10081572, 'Inappropriate schedule of product administration'; both strings were included in P1. Frozen strings absent from v29.0 contributed no reports. | Assesses terminology drift; cannot exclude period-related changes in coding practice. |

Definitions are report-level operational rules. Preferred terms were mapped using the prespecified frozen dictionary. The table states the data window, primary-suspect exposure restriction, version/deletion handling, and terminology-version boundary.

**Table S2. Expanded report characteristics, completeness, annual reporting process, and reports outside the active comparison**

| **Panel** | **Characteristic or year** | **Total n** | **F1 value** | **I1 value** | **Process indicator or statistic** | **Notes** |
| --- | --- | --- | --- | --- | --- | --- |
| **Panel A. Report characteristics and completeness** | Age, years: Median [IQR]; available n/N | — | 53.0 [48.0, 60.0]; 213/445 | 57.0 [50.0, 62.0]; 465/834 | SMD -0.264 | Descriptive comparison within the active F1/I1 set. |
|  | Age group: <65 | — | 192 (43.1%) | 398 (47.7%) | SMD -0.092 | Descriptive comparison within the active F1/I1 set. |
|  | Age group: 65-74 | — | 19 (4.3%) | 63 (7.6%) | SMD -0.140 | Descriptive comparison within the active F1/I1 set. |
|  | Age group: ≥75 | — | 2 (0.4%) | 4 (0.5%) | SMD -0.004 | Descriptive comparison within the active F1/I1 set. |
|  | Age group: Missing | — | 232 (52.1%) | 369 (44.2%) | SMD 0.158 | Descriptive comparison within the active F1/I1 set. |
|  | Healthcare-professional reporter: Yes | — | 228 (51.2%) | 195 (23.4%) | SMD 0.601 | Descriptive comparison within the active F1/I1 set. |
|  | Manufacturer-origin report: Yes | — | 441 (99.1%) | 831 (99.6%) | SMD -0.068 | Descriptive comparison within the active F1/I1 set. |
|  | United States report: Yes | — | 398 (89.4%) | 824 (98.8%) | SMD -0.406 | Descriptive comparison within the active F1/I1 set. |
|  | Literature report: Yes | — | 52 (11.7%) | 2 (0.2%) | SMD 0.498 | Descriptive comparison within the active F1/I1 set. |
|  | Procedural/use composite (P1): Yes | — | 57 (12.8%) | 171 (20.5%) | SMD -0.208 | Descriptive comparison within the active F1/I1 set. |
|  | Anticoagulant co-report: Yes | — | 0 (0.0%) | 8 (1.0%) | SMD -0.139 | Descriptive comparison within the active F1/I1 set. |
|  | Antiplatelet co-report: Yes | — | 13 (2.9%) | 42 (5.0%) | SMD -0.108 | Descriptive comparison within the active F1/I1 set. |
|  | PDE5 inhibitor co-report: Yes | — | 22 (4.9%) | 38 (4.6%) | SMD 0.018 | Descriptive comparison within the active F1/I1 set. |
|  | Surgery/repair phenotype (M1): Yes | — | 194 (43.6%) | 30 (3.6%) | SMD 1.068 | Descriptive comparison within the active F1/I1 set. |
|  | Haematoma/haemorrhage phenotype (H1): Yes | — | 36 (8.1%) | 653 (78.3%) | SMD -2.009 | Descriptive comparison within the active F1/I1 set. |
|  | Functional impairment phenotype (S1): Yes | — | 7 (1.6%) | 45 (5.4%) | SMD -0.210 | Descriptive comparison within the active F1/I1 set. |
|  | Hospitalization code: Yes | — | 71 (16.0%) | 29 (3.5%) | SMD 0.431 | Descriptive comparison within the active F1/I1 set. |
|  | Report period: 2014-2017 | — | 140 (31.5%) | 241 (28.9%) | SMD 0.056 | Descriptive comparison within the active F1/I1 set. |
|  | Report period: 2018-2021 | — | 178 (40.0%) | 475 (57.0%) | SMD -0.344 | Descriptive comparison within the active F1/I1 set. |
|  | Report period: 2022-2025 | — | 127 (28.5%) | 118 (14.1%) | SMD 0.357 | Descriptive comparison within the active F1/I1 set. |
|  | Any serious outcome code: Yes | — | 445 (100.0%) | 168 (20.1%) | SMD 2.816 | Shown descriptively because serious-outcome coding was empirically invariant among F1 reports in this cohort; it was not modeled as an independent discriminator. |
|  | Age available | — | 213 (47.9%) | 465 (55.8%) | Availability | Availability is reported, not missingness. |
|  | Event date complete | — | 167 (37.5%) | 345 (41.4%) | Availability | Availability is reported, not missingness. |
|  | Usable therapy-start-to-event interval | — | 91 (20.4%) | Not analysed | Availability | Availability is reported, not missingness. |
|  | Weight available | — | 113 (25.4%) | 426 (51.1%) | Availability | Availability is reported, not missingness. |
| **Panel B. Annual reporting process** | 2014 | 14 | 8 | 6 | HCP 92.9%; literature 0.0%; age known 100.0%; usable F1 interval 50.0% | Percentages use all active-comparison reports in the year, except usable therapy-start-to-event interval, which uses F1 reports. |
|  | 2015 | 129 | 24 | 105 | HCP 49.6%; literature 0.8%; age known 69.8%; usable F1 interval 29.2% | Percentages use all active-comparison reports in the year, except usable therapy-start-to-event interval, which uses F1 reports. |
|  | 2016 | 120 | 54 | 66 | HCP 54.2%; literature 9.2%; age known 65.0%; usable F1 interval 18.5% | Percentages use all active-comparison reports in the year, except usable therapy-start-to-event interval, which uses F1 reports. |
|  | 2017 | 118 | 54 | 64 | HCP 38.1%; literature 0.0%; age known 57.6%; usable F1 interval 20.4% | Percentages use all active-comparison reports in the year, except usable therapy-start-to-event interval, which uses F1 reports. |
|  | 2018 | 124 | 49 | 75 | HCP 16.1%; literature 0.0%; age known 55.6%; usable F1 interval 26.5% | Percentages use all active-comparison reports in the year, except usable therapy-start-to-event interval, which uses F1 reports. |
|  | 2019 | 182 | 54 | 128 | HCP 20.3%; literature 0.0%; age known 58.2%; usable F1 interval 24.1% | Percentages use all active-comparison reports in the year, except usable therapy-start-to-event interval, which uses F1 reports. |
|  | 2020 | 223 | 45 | 178 | HCP 17.9%; literature 1.3%; age known 50.2%; usable F1 interval 31.1% | Percentages use all active-comparison reports in the year, except usable therapy-start-to-event interval, which uses F1 reports. |
|  | 2021 | 124 | 30 | 94 | HCP 24.2%; literature 0.8%; age known 39.5%; usable F1 interval 33.3% | Percentages use all active-comparison reports in the year, except usable therapy-start-to-event interval, which uses F1 reports. |
|  | 2022 | 77 | 18 | 59 | HCP 5.2%; literature 0.0%; age known 40.3%; usable F1 interval 0.0% | Percentages use all active-comparison reports in the year, except usable therapy-start-to-event interval, which uses F1 reports. |
|  | 2023 | 31 | 16 | 15 | HCP 38.7%; literature 3.2%; age known 41.9%; usable F1 interval 18.8% | Percentages use all active-comparison reports in the year, except usable therapy-start-to-event interval, which uses F1 reports. |
|  | 2024 | 65 | 48 | 17 | HCP 75.4%; literature 18.5%; age known 36.9%; usable F1 interval 8.3% | Percentages use all active-comparison reports in the year, except usable therapy-start-to-event interval, which uses F1 reports. |
|  | 2025 | 72 | 45 | 27 | HCP 61.1%; literature 34.7%; age known 33.3%; usable F1 interval 4.4% | Percentages use all active-comparison reports in the year, except usable therapy-start-to-event interval, which uses F1 reports. |
| **Panel C. CCH reports outside the active F1/I1 comparison** | Total reports meeting neither F1 nor I1 | 2,379 | Not applicable | Not applicable | 2379 (100.0%) | These reports met the CCH primary-suspect and strict-indication rules but contained neither a frozen F1 term nor an I1 qualifying term; they were not excluded because of missing patient data. |
|  | Report period: 2014–2017 | 2,379 | Not applicable | Not applicable | 413 (17.4%; 95% CI 15.9–18.9) | Denominator is the 2,379 CCH reports meeting neither F1 nor I1. |
|  | Report period: 2018–2021 | 2,379 | Not applicable | Not applicable | 1179 (49.6%; 95% CI 47.5–51.6) | Denominator is the 2,379 CCH reports meeting neither F1 nor I1. |
|  | Report period: 2022–2025 | 2,379 | Not applicable | Not applicable | 787 (33.1%; 95% CI 31.2–35.0) | Denominator is the 2,379 CCH reports meeting neither F1 nor I1. |
|  | M1 plus hospitalization coding | 2,379 | Not applicable | Not applicable | 2 (0.1%; 95% CI 0.0–0.3) | Denominator is the 2,379 CCH reports meeting neither F1 nor I1. |
|  | M1 surgery/repair coding | 2,379 | Not applicable | Not applicable | 6 (0.3%; 95% CI 0.1–0.5) | Denominator is the 2,379 CCH reports meeting neither F1 nor I1. |
|  | Hospitalization coding | 2,379 | Not applicable | Not applicable | 65 (2.7%; 95% CI 2.1–3.5) | Denominator is the 2,379 CCH reports meeting neither F1 nor I1. |

Panel A reports descriptive characteristics and field completeness; Panel B reports annual process indicators; Panel C characterizes the 2,379 CCH primary-suspect strict-indication reports meeting neither F1 nor I1. Those reports were outside the active phenotype contrast, not excluded for missing patient data. Annual HCP, literature, and age-known percentages use the active F1/I1 set; usable interval percentages use F1 reports in the corresponding year.

**Table S3. Complete primary, sensitivity, and diagnostic model results**

| **Section** | **Model or term** | **Analysis n** | **Adjusted estimate (95% CI)** | **P value** | **Diagnostic or status** | **Adjustment set** |
| --- | --- | --- | --- | --- | --- | --- |
| **Deletion-aware primary model: complete coefficients** | Healthcare-professional reporter | 1,279 | 3.24 (2.50–4.21) | <0.001 | Firth logistic regression; profile-likelihood CI | Report period + healthcare-professional reporter + manufacturer origin + P1 |
|  | Manufacturer-origin report | 1,279 | 0.51 (0.11–2.19) | 0.359 | Firth logistic regression; profile-likelihood CI; sparse manufacturer-origin variation | Report period + healthcare-professional reporter + manufacturer origin + P1 |
|  | 2018–2021 vs 2014–2017 | 1,279 | 0.94 (0.70–1.26) | 0.676 | Firth logistic regression; profile-likelihood CI | Report period + healthcare-professional reporter + manufacturer origin + P1 |
|  | 2022–2025 vs 2014–2017 | 1,279 | 2.22 (1.58–3.15) | <0.001 | Firth logistic regression; profile-likelihood CI | Report period + healthcare-professional reporter + manufacturer origin + P1 |
|  | Procedural/use composite (P1) | 1,279 | 0.50 (0.35–0.71) | <0.001 | Firth logistic regression; profile-likelihood CI | Report period + healthcare-professional reporter + manufacturer origin + P1 |
| **Alternative comparator, restriction, and reporting-process models** | Primary: all I1 | 1,279 | 0.50 (0.35–0.71) | <0.001 | Primary | Report period + healthcare-professional reporter + manufacturer origin + P1 |
|  | I1-H bleeding/contusion control | 1,098 | 0.51 (0.35–0.74) | <0.001 | Comparator | Report period + healthcare-professional reporter + manufacturer origin + P1 |
|  | I1-P pain/swelling control | 1,048 | 0.48 (0.33–0.69) | <0.001 | Comparator | Report period + healthcare-professional reporter + manufacturer origin + P1 |
|  | Healthcare-professional reports | 423 | 0.53 (0.29–0.95) | 0.032 | Restriction | Report period + manufacturer origin + P1 |
|  | Missing-category age adjustment | 1,279 | 0.54 (0.38–0.76) | <0.001 | Missingness | Report period + healthcare-professional reporter + manufacturer origin + age group + P1 |
|  | Age-complete cases + age group | 678 | 0.72 (0.46–1.11) | 0.140 | Missingness | Report period + healthcare-professional reporter + manufacturer origin + age group + P1 |
|  | All non-F1 reports as controls | 3,635 | 0.81 (0.59–1.09) | 0.163 | Comparator | Report period + healthcare-professional reporter + manufacturer origin + P1 |
|  | Decontaminated report-richness model | 1,279 | 0.41 (0.27–0.61) | <0.001 | Reporting process | Report period + healthcare-professional reporter + manufacturer origin + literature report + United States report + log(non-index preferred-term count) + log(drug count) + P1 |
| **Independent bias-reduced model cross-check** | Six prespecified severity and deletion-sensitivity models | — | Firth and independent ORs agreed to three decimals | — | All models converged; direction concordant; maximum relative difference <0.001% | Corresponding Firth-model design matrices |

Primary and sensitivity models use Firth logistic regression. Independent bias-reduced fits are verification checks. Manufacturer-origin reporting has sparse variation and its coefficient is correspondingly imprecise. CI, confidence interval.

**Table S4. P1 subdomain frequencies and robustness analyses**

| **Section** | **Domain, removed term, or period** | **F1 events / N** | **I1 events / N** | **Model** | **Adjusted reporting odds ratio (95% CI)** | **P value** | **Notes** |
| --- | --- | --- | --- | --- | --- | --- | --- |
| **P1 subdomain frequency** | Treatment nonadherence | 22/445 (4.9%; 95% CI 3.1–7.4) | 5/834 (0.6%; 95% CI 0.2–1.4) | Descriptive | — | — | Exact binomial 95% CI. |
|  | Schedule/dose/duration | 19/445 (4.3%; 95% CI 2.6–6.6) | 112/834 (13.4%; 95% CI 11.2–15.9) | Descriptive | — | — | Exact binomial 95% CI. |
|  | Technique/site | 3/445 (0.7%; 95% CI 0.1–2.0) | 55/834 (6.6%; 95% CI 5.0–8.5) | Descriptive | — | — | Exact binomial 95% CI. |
|  | Unapproved/intentional/general use | 17/445 (3.8%; 95% CI 2.2–6.0) | 15/834 (1.8%; 95% CI 1.0–2.9) | Descriptive | — | — | Exact binomial 95% CI. |
| **P1 subdomain model** | Technique/site | See frequency rows | See frequency rows | Base-adjusted | 0.12 (0.03–0.31) | <0.001 | Deletion-aware Firth model; nominal P value; prespecified exploratory domain analysis. |
|  | Schedule/dose/duration | See frequency rows | See frequency rows | Base-adjusted | 0.32 (0.18–0.52) | <0.001 | Deletion-aware Firth model; nominal P value; prespecified exploratory domain analysis. |
|  | Treatment nonadherence | See frequency rows | See frequency rows | Base-adjusted | 5.27 (2.08–15.69) | <0.001 | Deletion-aware Firth model; nominal P value; prespecified exploratory domain analysis. |
|  | Unapproved/intentional/general use | See frequency rows | See frequency rows | Base-adjusted | 1.35 (0.62–2.95) | 0.441 | Deletion-aware Firth model; nominal P value; prespecified exploratory domain analysis. |
|  | Technique/site | See frequency rows | See frequency rows | Mutually adjusted | 0.12 (0.03–0.33) | <0.001 | Deletion-aware Firth model; nominal P value; prespecified exploratory domain analysis. |
|  | Schedule/dose/duration | See frequency rows | See frequency rows | Mutually adjusted | 0.34 (0.20–0.56) | <0.001 | Deletion-aware Firth model; nominal P value; prespecified exploratory domain analysis. |
|  | Treatment nonadherence | See frequency rows | See frequency rows | Mutually adjusted | 5.72 (2.16–17.79) | <0.001 | Deletion-aware Firth model; nominal P value; prespecified exploratory domain analysis. |
|  | Unapproved/intentional/general use | See frequency rows | See frequency rows | Mutually adjusted | 1.56 (0.71–3.46) | 0.266 | Deletion-aware Firth model; nominal P value; prespecified exploratory domain analysis. |
| **Leave-one-preferred-term-out analysis** | treatment noncompliance | — | — | Removed term present in 27 reports | 0.34 (0.23–0.50) | <0.001 | P1 composite reconstructed after removing one preferred term. |
|  | off label use | — | — | Removed term present in 23 reports | 0.42 (0.29–0.61) | <0.001 | P1 composite reconstructed after removing one preferred term. |
|  | inappropriate schedule of drug administration | — | — | Removed term present in 39 reports | 0.49 (0.33–0.71) | <0.001 | P1 composite reconstructed after removing one preferred term. |
|  | product use in unapproved indication | — | — | Removed term present in 0 reports | 0.50 (0.35–0.71) | <0.001 | P1 composite reconstructed after removing one preferred term. |
|  | product use issue | — | — | Removed term present in 1 reports | 0.51 (0.35–0.71) | <0.001 | P1 composite reconstructed after removing one preferred term. |
|  | intentional product use issue | — | — | Removed term present in 8 reports | 0.51 (0.36–0.72) | <0.001 | P1 composite reconstructed after removing one preferred term. |
|  | product administered at inappropriate site | — | — | Removed term present in 4 reports | 0.51 (0.36–0.72) | <0.001 | P1 composite reconstructed after removing one preferred term. |
|  | product administration error | — | — | Removed term present in 6 reports | 0.51 (0.36–0.72) | <0.001 | P1 composite reconstructed after removing one preferred term. |
|  | incorrect product administration duration | — | — | Removed term present in 9 reports | 0.52 (0.37–0.74) | <0.001 | P1 composite reconstructed after removing one preferred term. |
|  | incorrect dose administered | — | — | Removed term present in 6 reports | 0.53 (0.37–0.75) | <0.001 | P1 composite reconstructed after removing one preferred term. |
|  | inappropriate schedule of product administration | — | — | Removed term present in 78 reports | 0.64 (0.43–0.93) | 0.019 | P1 composite reconstructed after removing one preferred term. |
|  | wrong technique in product usage process | — | — | Removed term present in 50 reports | 0.66 (0.45–0.94) | 0.020 | P1 composite reconstructed after removing one preferred term. |
| **Calendar-period stratification** | 2014-2017 | — | — | Within-period deletion-aware model; n=381 | 0.20 (0.08–0.44) | <0.001 | Adjusted for healthcare-professional reporter and manufacturer origin. |
|  | 2018-2021 | — | — | Within-period deletion-aware model; n=653 | 0.41 (0.23–0.69) | <0.001 | Adjusted for healthcare-professional reporter and manufacturer origin. |
|  | 2022-2025 | — | — | Within-period deletion-aware model; n=245 | 0.95 (0.46–1.95) | 0.891 | Adjusted for healthcare-professional reporter and manufacturer origin. |
| **Calendar-period interaction** | P1 × report period | — | — | Penalized likelihood-ratio test; df=2 | Not represented by a single OR | 0.135 | Global interaction test. |

All deletion-aware subdomain models include report period, healthcare-professional reporter, and manufacturer origin. Frequency rows show exact binomial 95% CIs. Domain-model P values are nominal and are not multiplicity-adjusted; these prespecified analyses are exploratory. Leave-one-term-out analyses reconstruct P1 after removing one preferred term.

**Table S5. Restricted coding ladder, hospitalization, and nested-outcome analyses**

| **Section** | **Level or contrast** | **n** | **P1, n (%) [95% CI]** | **Hospitalization, n (%) [95% CI]** | **Usable interval, n** | **Median interval [IQR], days** | **Adjusted ROR (95% CI)** | **P value** | **Notes** |
| --- | --- | --- | --- | --- | --- | --- | --- | --- | --- |
| **Mutually exclusive ladder description** | L0: I1 without M1 | 804 | 168 (20.9%) [18.1–23.9] | 20 (2.5%) [1.5–3.8] | 164 | 0.0 [0.0, 1.0] | — | — | Restricted coding rung; not a validated clinical grade. |
|  | L1: F1 without M1 | 251 | 33 (13.1%) [9.2–18.0] | 20 (8.0%) [4.9–12.0] | 37 | 5.0 [1.0, 23.0] | — | — | Restricted coding rung; not a validated clinical grade. |
|  | L2: F1 with M1 | 194 | 24 (12.4%) [8.1–17.8] | 51 (26.3%) [20.2–33.1] | 54 | 17.0 [3.2, 29.5] | — | — | Restricted coding rung; not a validated clinical grade. |
|  | I1 with M1 excluded | 30 | 3 (10.0%) [2.1–26.5] | 9 (30.0%) [14.7–49.4] | Not analysed | Not analysed | — | — | Excluded from the restricted ladder; retained descriptively because its 30% hospitalization proportion precludes a global severity ordering. |
| **Categorical hospitalization consistency model** | L1 versus L0 | 1,249 | — | See descriptive rows | — | — | 2.84 (1.43–5.62) | 0.003 | Outcome is hospitalization; L0 is the reference. Supports internal consistency only and does not validate clinical severity. |
|  | L2 versus L0 | 1,249 | — | See descriptive rows | — | — | 12.95 (7.51–23.16) | <0.001 | Outcome is hospitalization; L0 is the reference. Supports internal consistency only and does not validate clinical severity. |
| **Deletion-aware adjusted severity models** | F1 versus all I1 | 1,279 | — | — | — | — | 0.50 (0.35–0.71) | <0.001 | Predictor is P1. |
|  | F1 versus I1 without M1 | 1,249 | — | — | — | — | 0.48 (0.34–0.68) | <0.001 | Predictor is P1. |
|  | F1 with M1 versus F1 without M1 | 445 | — | — | — | — | 1.59 (0.85–3.00) | 0.146 | Predictor is P1. |
|  | Hospitalization per one-rung increase | 1,249 | — | — | — | — | 3.69 (2.80–4.94) | <0.001 | Outcome is hospitalization; predictor is one-rung ladder increase. |
|  | Hospitalization versus no hospitalization within F1 | 445 | — | — | — | — | 2.13 (0.99–4.40) | 0.052 | Predictor is P1. |
|  | F1+M1 versus all I1 | 1,028 | — | — | — | — | 0.56 (0.35–0.88) | 0.011 | Predictor is P1. |
|  | F1+hospitalization versus all I1 | 905 | — | — | — | — | 0.87 (0.44–1.62) | 0.669 | Predictor is P1. |
| **Nested and overlapping severe-outcome definitions** | Any F1 vs all I1 | 1,279 | — | — | — | — | 0.50 (0.35–0.71) | <0.001 | Holm-adjusted P=<0.001; rows are not mutually exclusive severity levels. |
|  | F1 + M1 vs all I1 | 1,028 | — | — | — | — | 0.56 (0.35–0.88) | 0.011 | Holm-adjusted P=0.032; rows are not mutually exclusive severity levels. |
|  | F1 + hospitalization vs all I1 | 905 | — | — | — | — | 0.87 (0.44–1.62) | 0.669 | Holm-adjusted P=1.000; rows are not mutually exclusive severity levels. |
|  | F1 + M1 + hospitalization vs all I1 | 885 | — | — | — | — | 0.77 (0.34–1.59) | 0.505 | Holm-adjusted P=1.000; rows are not mutually exclusive severity levels. |

L0, I1 without M1; L1, F1 without M1; L2, F1 with M1. I1 with M1 is excluded from the restricted ladder but retained descriptively. Categorical and linear hospitalization models assess supportive internal consistency only; they do not validate a global clinical severity scale. Adjusted models include report period, healthcare-professional reporter, and manufacturer origin; P1 is the predictor unless the row specifies the ladder score. Nested outcomes are not mutually exclusive, and Holm-adjusted P values are shown in the Notes column.

**Table S6. Therapy-start-to-event interval distribution and selection analysis**

| **Section** | **Measure or term** | **Count / denominator or analysis n** | **Result** | **Adjusted reporting odds ratio (95% CI)** | **P value** | **Notes** |
| --- | --- | --- | --- | --- | --- | --- |
| **Overall therapy-start-to-event summary** | F1 reports with a usable report-level interval | 91/445 | 20.4%; median 14.0 [1.0, 27.0] days; ≤28 days 71/91 | — | — | Report-level proxy; treatment-cycle boundaries and the most recent injection date are not reliably identified. This is not injection-to-fracture latency. |
| **Prespecified report-level interval** | 0–7 | 39/91 | 42.9% | — | — | Mutually exclusive window. |
|  | 8–14 | 7/91 | 7.7% | — | — | Mutually exclusive window. |
|  | 15–28 | 25/91 | 27.5% | — | — | Mutually exclusive window. |
|  | >28 | 20/91 | 22.0% | — | — | Mutually exclusive window. |
| **Cumulative therapy-start-to-event milestone** | ≤1 days | 24/91 | 26.4% [95% CI 17.7–36.7] | — | — | Exact binomial CI. |
|  | ≤7 days | 39/91 | 42.9% [95% CI 32.5–53.7] | — | — | Exact binomial CI. |
|  | ≤14 days | 46/91 | 50.5% [95% CI 39.9–61.2] | — | — | Exact binomial CI. |
|  | ≤28 days | 71/91 | 78.0% [95% CI 68.1–86.0] | — | — | Exact binomial CI. |
| **Therapy-start-to-event interval by M1 coding** | F1 without M1 | 37/251 | Median 5.0 [1.0, 23.0] days | — | — | Descriptive date-complete subset; not injection-to-fracture latency. |
|  | F1 with M1 | 54/194 | Median 17.0 [3.2, 29.5] days | — | — | Descriptive date-complete subset; not injection-to-fracture latency. |
| **Selection into the date-complete subset** | M1 surgery/repair coding | 445 | Outcome: usable therapy-start-to-event interval | 1.79 (0.97–3.35) | 0.063 | Firth model within deletion-aware F1 reports; describes interval availability but does not correct selection bias. |
|  | P1 procedural/use coding | 445 | Outcome: usable therapy-start-to-event interval | 0.25 (0.07–0.72) | 0.009 | Firth model within deletion-aware F1 reports; describes interval availability but does not correct selection bias. |
|  | 2018–2021 vs 2014–2017 | 445 | Outcome: usable therapy-start-to-event interval | 2.47 (1.26–5.01) | 0.008 | Firth model within deletion-aware F1 reports; describes interval availability but does not correct selection bias. |
|  | 2022–2025 vs 2014–2017 | 445 | Outcome: usable therapy-start-to-event interval | 0.49 (0.19–1.19) | 0.118 | Firth model within deletion-aware F1 reports; describes interval availability but does not correct selection bias. |
|  | Healthcare-professional reporter | 445 | Outcome: usable therapy-start-to-event interval | 2.56 (1.37–4.90) | 0.003 | Firth model within deletion-aware F1 reports; describes interval availability but does not correct selection bias. |
|  | Manufacturer-origin report | 445 | Outcome: usable therapy-start-to-event interval | 0.52 (0.06–6.57) | 0.569 | Firth model within deletion-aware F1 reports; describes interval availability but does not correct selection bias. |
|  | Age available | 445 | Outcome: usable therapy-start-to-event interval | 25.28 (10.78–69.17) | <0.001 | Firth model within deletion-aware F1 reports; describes interval availability but does not correct selection bias. |
|  | Log(1 + PT count) | 445 | Outcome: usable therapy-start-to-event interval | 0.24 (0.09–0.60) | 0.002 | Firth model within deletion-aware F1 reports; describes interval availability but does not correct selection bias. |
|  | Log(1 + drug count) | 445 | Outcome: usable therapy-start-to-event interval | 2.13 (1.26–3.66) | 0.004 | Firth model within deletion-aware F1 reports; describes interval availability but does not correct selection bias. |

The therapy-start-to-event interval is a report-level proxy among date-complete reports and is subject to substantial selection. FAERS does not reliably identify treatment-cycle boundaries or the latest injection date. The availability model characterizes entry into the date-complete subset but does not correct selection bias. Results are descriptive and not injection-to-fracture or patient-level latency estimates.

**Table S7. P1 tipping-point and deletion-list sensitivity**

| **Section** | **Dataset** | **Scenario or model** | **k** | **Reclassified F1 reports** | **Crude result** | **Adjusted estimate or simulation summary** | **Crude Fisher P** | **Fit status** | **Notes** |
| --- | --- | --- | --- | --- | --- | --- | --- | --- | --- |
| **Validated tipping threshold** | Original preprocessed analytic cohort | Crude prevalence-equality threshold | 35 | 7.8% of all F1; 9.0% of currently P1-negative F1 | One-way reclassification of P1-negative F1 reports | See checkpoint rows | — | — | Decision threshold from the prespecified analysis. |
|  | Original preprocessed analytic cohort | Crude Fisher P ≥ 0.05 threshold | 14 | 3.1% of all F1; 3.6% of currently P1-negative F1 | One-way reclassification of P1-negative F1 reports | See checkpoint rows | — | — | Decision threshold from the prespecified analysis. |
|  | Original preprocessed analytic cohort | Covariate-balanced adjusted statistical-null threshold | 20 | 4.5% of all F1; 5.1% of currently P1-negative F1 | One-way reclassification of P1-negative F1 reports | See checkpoint rows | — | — | The adjusted statistical-null threshold is the first k for which the balanced 95% CI includes 1. |
|  | Original preprocessed analytic cohort | Covariate-balanced adjusted point-null threshold | 41 | 9.2% of all F1; 10.5% of currently P1-negative F1 | One-way reclassification of P1-negative F1 reports | See checkpoint rows | — | — | Decision threshold from the prespecified analysis. |
|  | Deletion-list-aware analytic cohort | Crude prevalence-equality threshold | 35 | 7.9% of all F1; 9.0% of currently P1-negative F1 | One-way reclassification of P1-negative F1 reports | See checkpoint rows | — | — | Decision threshold from the prespecified analysis. |
|  | Deletion-list-aware analytic cohort | Crude Fisher P ≥ 0.05 threshold | 14 | 3.1% of all F1; 3.6% of currently P1-negative F1 | One-way reclassification of P1-negative F1 reports | See checkpoint rows | — | — | Decision threshold from the prespecified analysis. |
|  | Deletion-list-aware analytic cohort | Covariate-balanced adjusted statistical-null threshold | 20 | 4.5% of all F1; 5.2% of currently P1-negative F1 | One-way reclassification of P1-negative F1 reports | See checkpoint rows | — | — | The adjusted statistical-null threshold is the first k for which the balanced 95% CI includes 1. |
|  | Deletion-list-aware analytic cohort | Covariate-balanced adjusted point-null threshold | 41 | 9.2% of all F1; 10.6% of currently P1-negative F1 | One-way reclassification of P1-negative F1 reports | See checkpoint rows | — | — | Decision threshold from the prespecified analysis. |
| **Decision-relevant tipping-path checkpoint** | Original preprocessed analytic cohort | Observed | 0 | 0.0% of all F1; 0.0% of P1-negative F1 | OR 0.568 | 0.50 (0.35–0.71) | <0.001 | Yes | Covariate-balanced deterministic allocation; adjusted inference is based on the 95% CI. |
|  | Original preprocessed analytic cohort | Crude Fisher null | 14 | 3.1% of all F1; 3.6% of P1-negative F1 | OR 0.734 | 0.66 (0.48–0.92) | 0.051 | Yes | Covariate-balanced deterministic allocation; adjusted inference is based on the 95% CI. |
|  | Original preprocessed analytic cohort | Adjusted statistical null | 20 | 4.5% of all F1; 5.1% of P1-negative F1 | OR 0.809 | 0.73 (0.53–1.01) | 0.182 | Yes | Covariate-balanced deterministic allocation; adjusted inference is based on the 95% CI. |
|  | Original preprocessed analytic cohort | Crude prevalence equality | 35 | 7.8% of all F1; 9.0% of P1-negative F1 | OR 1.008 | 0.93 (0.68–1.26) | 1.000 | Yes | Covariate-balanced deterministic allocation; adjusted inference is based on the 95% CI. |
|  | Original preprocessed analytic cohort | Adjusted point null | 41 | 9.2% of all F1; 10.5% of P1-negative F1 | OR 1.092 | 1.01 (0.75–1.36) | 0.565 | Yes | Covariate-balanced deterministic allocation; adjusted inference is based on the 95% CI. |
|  | Deletion-list-aware analytic cohort | Observed | 0 | 0.0% of all F1; 0.0% of P1-negative F1 | OR 0.570 | 0.50 (0.36–0.71) | <0.001 | Yes | Covariate-balanced deterministic allocation; adjusted inference is based on the 95% CI. |
|  | Deletion-list-aware analytic cohort | Crude Fisher null | 14 | 3.1% of all F1; 3.6% of P1-negative F1 | OR 0.736 | 0.66 (0.48–0.92) | 0.051 | Yes | Covariate-balanced deterministic allocation; adjusted inference is based on the 95% CI. |
|  | Deletion-list-aware analytic cohort | Adjusted statistical null | 20 | 4.5% of all F1; 5.2% of P1-negative F1 | OR 0.811 | 0.74 (0.53–1.01) | 0.182 | Yes | Covariate-balanced deterministic allocation; adjusted inference is based on the 95% CI. |
|  | Deletion-list-aware analytic cohort | Crude prevalence equality | 35 | 7.9% of all F1; 9.0% of P1-negative F1 | OR 1.010 | 0.93 (0.69–1.26) | 0.942 | Yes | Covariate-balanced deterministic allocation; adjusted inference is based on the 95% CI. |
|  | Deletion-list-aware analytic cohort | Adjusted point null | 41 | 9.2% of all F1; 10.6% of P1-negative F1 | OR 1.095 | 1.01 (0.75–1.36) | 0.565 | Yes | Covariate-balanced deterministic allocation; adjusted inference is based on the 95% CI. |
| **Random-allocation uncertainty** | Deletion-list-aware analytic cohort | Adjusted statistical-null checkpoint | 20 | 4.5% of all F1; 5.2% of P1-negative F1 | — | Random-allocation median OR 0.74; allocation 2.5th–97.5th percentiles 0.71–0.76; median 95% CI 0.54–1.01 | — | 100.0% | 1,000 random allocations without replacement; 73.7% of fitted 95% CIs included 1 and 0.0% of point estimates were ≥1; seed 20260808. |
|  | Deletion-list-aware analytic cohort | Crude prevalence-equality checkpoint | 35 | 7.9% of all F1; 9.0% of P1-negative F1 | — | Random-allocation median OR 0.93; allocation 2.5th–97.5th percentiles 0.89–0.97; median 95% CI 0.69–1.26 | — | 100.0% | 1,000 random allocations without replacement; 100.0% of fitted 95% CIs included 1 and 0.2% of point estimates were ≥1; seed 20260808. |
|  | Deletion-list-aware analytic cohort | Adjusted point-null checkpoint | 41 | 9.2% of all F1; 10.6% of P1-negative F1 | — | Random-allocation median OR 1.01; allocation 2.5th–97.5th percentiles 0.97–1.06; median 95% CI 0.75–1.37 | — | 100.0% | 1,000 random allocations without replacement; 100.0% of fitted 95% CIs included 1 and 70.7% of point estimates were ≥1; seed 20260808. |
| **Deletion-list count sensitivity** | Original preprocessed analytic cohort | Cohort counts | — | All target reports 3,637; active comparison 1,280; F1 446; I1 834 | F1+M1 195; F1 hospitalization 71 | — | — | — | Removed reports=2; removed F1=1; removed M1=1; removed P1=0; removed hospitalization=0 |
|  | Deletion-list-aware analytic cohort | Cohort counts | — | All target reports 3,635; active comparison 1,279; F1 445; I1 834 | F1+M1 194; F1 hospitalization 71 | — | — | — | Removed reports=2; removed F1=1; removed M1=1; removed P1=0; removed hospitalization=0 |
| **Deletion-list model sensitivity** | Original preprocessed analytic cohort | Any F1 vs all I1: P1 association | — | — | — | 0.50 (0.35–0.71); P <0.001 | — | Yes | Firth logistic regression with profile-likelihood CI. |
|  | Original preprocessed analytic cohort | Hospitalization per severity-ladder rung | — | — | — | 3.67 (2.78–4.92); P <0.001 | — | Yes | Firth logistic regression with profile-likelihood CI. |
|  | Deletion-list-aware analytic cohort | Any F1 vs all I1: P1 association | — | — | — | 0.50 (0.35–0.71); P <0.001 | — | Yes | Firth logistic regression with profile-likelihood CI. |
|  | Deletion-list-aware analytic cohort | Hospitalization per severity-ladder rung | — | — | — | 3.69 (2.80–4.94); P <0.001 | — | Yes | Firth logistic regression with profile-likelihood CI. |

k denotes additional currently P1-negative F1 reports reclassified as P1-positive. Crude Fisher P values are shown only for the crude path; deterministic adjusted tipping inference is based on whether the balanced-model 95% CI includes 1. Random-allocation rows summarize 1,000 allocations without replacement at each reported checkpoint. Thresholds evaluate robustness to one-way differential under-recording.

**Table S8. Same-indication comparator feasibility and data reconciliation**

| **Section** | **Item** | **Reports / sample** | **F1 events / estimate** | **Status or result** | **Interpretation** |
| --- | --- | --- | --- | --- | --- |
| **Same-indication comparator decision** | Overall decision | 54 non-CCH primary-suspect reports across heterogeneous therapies | 0 F1 events | Not feasible | No coherent comparator met the prespecified minimum rule; no coherent comparator model was forced. |
| **Same-indication comparator feasibility** | Interferon alfa-2b | 14 | 0 | Not feasible: <50 comparator reports | Not feasible: fewer than 50 comparator reports and/or fewer than five F1 events; no comparative model fitted. |
|  | fibrinogen human\human thrombin | 9 | 0 | Not feasible: <50 comparator reports | Not feasible: fewer than 50 comparator reports and/or fewer than five F1 events; no comparative model fitted. |
|  | Tamoxifen | 7 | 0 | Not feasible: <50 comparator reports | Not feasible: fewer than 50 comparator reports and/or fewer than five F1 events; no comparative model fitted. |
|  | Tadalafil | 6 | 0 | Not feasible: <50 comparator reports | Not feasible: fewer than 50 comparator reports and/or fewer than five F1 events; no comparative model fitted. |
|  | Sildenafil | 3 | 0 | Not feasible: <50 comparator reports | Not feasible: fewer than 50 comparator reports and/or fewer than five F1 events; no comparative model fitted. |
|  | Verapamil | 3 | 0 | Not feasible: <50 comparator reports | Not feasible: fewer than 50 comparator reports and/or fewer than five F1 events; no comparative model fitted. |
|  | Alprostadil | 2 | 0 | Not feasible: <50 comparator reports | Not feasible: fewer than 50 comparator reports and/or fewer than five F1 events; no comparative model fitted. |
|  | Pentoxifylline | 1 | 0 | Not feasible: <50 comparator reports | Not feasible: fewer than 50 comparator reports and/or fewer than five F1 events; no comparative model fitted. |
|  | Triamcinolone | 1 | 0 | Not feasible: <50 comparator reports | Not feasible: fewer than 50 comparator reports and/or fewer than five F1 events; no comparative model fitted. |
|  | deflazacort | 1 | 0 | Not feasible: <50 comparator reports | Not feasible: fewer than 50 comparator reports and/or fewer than five F1 events; no comparative model fitted. |
| **Quarterly-archive/preprocessed reconciliation** | Original preprocessed cohort | 3,637 | F1 446; I1 834; M1 231 | Reconciled | Reconstructed from the original preprocessed files using the strict indication, linked primary-suspect role, and target-therapy brand/ingredient confirmation. |
|  | Latest-version quarterly-archive CCH primary-suspect cohort | 3,658 | F1 445; I1 834; M1 230 | Reconciled | Retained the latest report version and excluded reports listed in FDA deleted-case files. |
|  | Reports present only in the original preprocessed cohort | 2 | F1 1; I1 0; M1 1 | Reconciled | Present in the original preprocessed cohort but absent after complete version and deletion-list handling. |
|  | Original-only reports appearing on FDA deletion lists | 2 | F1 1; I1 0; M1 1 | Reconciled | Both report identifiers appeared in FDA deleted-case files. |
|  | Reports identified only in the complete quarterly-archive scan | 23 | F1 0; I1 0; M1 0 | Reconciled | Qualifying target-therapy primary-suspect reports identified only in the complete quarterly-archive scan; none carried F1, I1, or M1 coding. |

Comparator modelling required at least 50 reports and five F1 events. No coherent comparator met this rule. Reconciliation results are aggregate and contain no report identifiers. Independent model verification is reported once in Table S3.
