# Supplementary material for: The reporting phenotype of penile fracture after collagenase clostridium histolyticum for Peyronie’s disease: an indication-restricted FAERS study
Source: Front Pharmacol. 2026 Oct 14;17:1965417. doi: 10.3389/fphar.2026.1965417 (PMC13713637; doi:10.3389/fphar.2026.1965417)
Supplement: Supplementary file 2 [file Supplementaryfile2.docx]

Supplementary File S2

# FAERS-specific study protocol

This document is an English, manuscript-specific translation and extract of protocol dated 07 July 2026. Translation and formatting were performed for peer-review accessibility. Subsequent analytic additions are identified with dates in Supplementary File S3.

## 1. Scientific position

Corporal rupture and severe penile injury are established complications of collagenase clostridium histolyticum (CCH) therapy for Peyronie’s disease. The study is not designed to discover a new risk or estimate incidence. Its purpose is to characterize how a known serious risk is represented in spontaneous reports after strict product, indication, drug-role, version, and deletion-list restriction.

All study estimates are reporting proportions, co-coding measures, or reporting odds. They are not patient-level risk estimates, treatment-effect estimates, or causal effects.

## 2. Primary objectives

1. Construct a strict CCH–Peyronie’s disease cohort from all FAERS quarterly archives from 2014Q1 through 2025Q4.
2. Compare fracture-coded reports with mutually exclusive non-fracture local-injury reports within the same indication and target therapy.
3. Describe co-recording of surgery/repair, hospitalization, functional impairment, hematoma/hemorrhage, and procedural or product-use details.
4. Assess the extent to which reporting period, reporter type, report source, geography, and information completeness shape the observed reporting phenotype.
5. Describe the available report-level timing proxy without interpreting it as an injection-specific hazard or patient-level time-to-event outcome.
6. Place the report phenotype in clinical context using regulatory information, pivotal or pooled clinical-development evidence, and a chart-reviewed clinical cohort without pooling incompatible denominators.

## 3. Data source and study period

The source is the FDA Adverse Event Reporting System ASCII quarterly files. All DEMO, DRUG, INDI, REAC, OUTC, RPSR, and therapy-date information required for the prespecified analyses is reconstructed at report level.

The data window is 2014Q1–2025Q4. Quarterly files are treated as versioned submissions rather than independent patients. The database does not provide a denominator of exposed or treated patients.

## 4. Report reconstruction and deletion handling

Candidate reports are identified by strict Peyronie’s disease indication variants. All DEMO versions for each candidate CASEID are rescanned. The highest CASEVERSION is retained, with FDA receipt date and PRIMARYID used as sequential tie-breakers when required. The retained version must still contain the strict indication. Reports appearing in FDA deleted-case files are excluded.

The analysis unit is the retained case-level report, not an individual reaction row or drug row. Aggregate deliverables must not contain report identifiers or narratives.

## 5. Product, indication, and drug-role restriction

CCH exposure is identified from normalized drug names, active-ingredient fields, and explicit XIAFLEX or XIAPEX product names. The strict indication is the MedDRA concept Peyronie’s disease; penile curvature alone is insufficient.

Eligibility requires a CCH drug sequence coded as primary suspect and linked to the strict indication. All primary-suspect sequences are enumerated. Reports in which CCH appears only as secondary suspect, concomitant, or interacting are excluded.

## 6. Prespecified report phenotypes

### 6.1 F1 fracture-coded phenotype

F1 requires the exact MedDRA preferred term *Fracture of penis*. F1 is a coding phenotype and does not establish chart-adjudicated corporal rupture.

### 6.2 I1 active local-injury comparator

I1 requires absence of F1 and at least one frozen penile or genital local-injury term covering hematoma, hemorrhage, contusion, swelling, edema, pain, injury, or laceration. F1 and I1 are mutually exclusive.

### 6.3 M1 surgery/repair coding

M1 requires at least one frozen surgery or repair term, including corpora cavernosa surgery, penile operation, or penile surgery. M1 is a co-reported management attribute and does not prove that an operation was completed or linked to a specific reaction.

### 6.4 Hospitalization

Hospitalization is defined from the FAERS outcome code for hospitalization or prolonged hospitalization. Outcome coding lacks consistent reaction-level linkage.

### 6.5 P1 procedural/use reporting summary

P1 is a high-sensitivity indicator that a report contains at least one of 12 frozen procedural, use, schedule, dose, duration, adherence, or intentional/general-use terms. It is not a single clinical exposure or mechanism. Four non-exclusive domains are prespecified: technique/site; schedule/dose/duration; treatment nonadherence; and unapproved/intentional/general use.

## 7. Analysis sets

- AS1: all deletion-list-aware, latest-version, strict-indication CCH primary-suspect reports.
- AS2: mutually exclusive F1 and I1 reports within AS1.
- Restricted coding ladder: L0, I1 without M1; L1, F1 without M1; L2, F1 with M1. I1 with M1 is retained descriptively and excluded from the restricted order.
- Date-complete F1 subset: F1 reports with a usable non-negative therapy-start-to-event interval.

## 8. Reporting-process variables

Core reporting-process variables are report period, healthcare-professional reporter, and manufacturer origin. Literature source, US origin, age availability, preferred-term count, drug count, and selected concomitant-drug indicators are reserved for prespecified descriptive or sensitivity analyses.

Age is converted to years. Values outside 0–120 years are set to missing. Missing age is not multiply imputed; complete-case and missing-category sensitivity analyses are used.

## 9. Primary estimand and statistical approach

The principal model-based estimand is the reporting odds ratio for P1 in F1 versus I1 reports after adjustment for the fixed core reporting-process variables. Firth penalized logistic regression is used because of sparse cells and possible separation. Profile-likelihood 95% confidence intervals and two-sided P values are reported.

Observed counts, proportions, standardized mean differences, and exact binomial confidence intervals are reported independently of the regression models. Model estimates are not interpreted as incidence, risk ratios, causal effects, or protection.

## 10. Secondary and sensitivity analyses

Prespecified analyses include:

7. P1 domain-specific models and a mutually adjusted four-domain model.
8. Leave-one-preferred-term-out reconstruction of P1.
9. Calendar-period-specific estimates and a P1-by-period interaction test.
10. Alternative non-fracture local-injury comparator subsets.
11. Healthcare-professional-only and age sensitivity models.
12. An expanded reporting-process model including literature source, US origin, non-index preferred-term count, and drug count.
13. Within-F1 comparisons involving M1 and hospitalization.
14. A quantitative one-way P1 under-recording tipping-point analysis.
15. A conditional same-indication comparator scan with minimum report and event thresholds.

## 11. Report-level timing

For each qualifying target-drug sequence with exact treatment-start and event dates, a non-negative therapy-start-to-event interval is calculated. When more than one qualifying sequence is present, every interval is evaluated and the shortest non-negative interval is retained.

The treatment-start date may not identify the latest injection in a repeated-cycle regimen. The interval is therefore descriptive and cannot be interpreted as injection-to-fracture latency. Selection into the date-complete subset is modeled as a reporting-process diagnostic but is not corrected by that model.

## 12. Clinical evidence triangulation

Regulatory labeling, pivotal or pooled clinical-development safety evidence, and a chart-reviewed Veterans Affairs cohort are compared qualitatively with the FAERS phenotype. Design, denominator, case definition, timing origin, and surgery/repair are recorded. Denominators are not pooled, and FAERS report proportions are not converted into clinical incidence.

## 13. Quality assurance and reproducibility

Core counts and model directions are independently cross-checked with a second mean-bias-reduction implementation. Statistical analysis scripts are supplied as Supplementary File S4, and the complete frozen operational and normalization dictionaries are supplied as Supplementary File S5. Software versions, model diagnostics, source manifests, and aggregate verification outputs are retained as internal quality-control records. Deliverables exclude report identifiers and narratives.

## 14. Interpretation boundaries

The following claims are prohibited:

- incidence or absolute risk;
- comparative treatment safety when no feasible comparator exists;
- patient-level risk prediction;
- causal attribution;
- clinical validation of a coding hierarchy;
- injection-to-fracture latency from the FAERS start date;
- a protective effect inferred from lower P1 recording.
