# Supplementary material for: The reporting phenotype of penile fracture after collagenase clostridium histolyticum for Peyronie’s disease: an indication-restricted FAERS study
Source: Front Pharmacol. 2026 Oct 14;17:1965417. doi: 10.3389/fphar.2026.1965417 (PMC13713637; doi:10.3389/fphar.2026.1965417)
Supplement: Supplementary file 3 [file Supplementaryfile3.docx]

Supplementary File S3

# FAERS-specific statistical analysis plan and dated amendment

This document consolidates only the analyses relevant to the present FAERS manuscript. The status of each analysis is stated explicitly.

## Part I. Base augmentation plan frozen on 09 July 2026

### 1. Fixed analysis sets and definitions

The existing AS1 strict CCH–Peyronie’s disease cohort, F1 fracture-coded phenotype, mutually exclusive I1 active local-injury comparator, M1 surgery/repair attribute, P1 procedural/use reporting summary, and core Firth specification were not redefined.

### 2. Outcome-definition robustness

Non-F1 comparator subdomains were prespecified as:

- bleeding/contusion;
- pain/swelling;
- structural injury.

For F1 versus each estimable domain, a Firth logistic model was specified with P1 as predictor and report period, healthcare-professional reporter, and manufacturer origin as adjustment covariates.

### 3. P1 decomposition and influence

P1 terms were grouped into four non-exclusive domains:

1. Technique/site.
2. Schedule/dose/duration.
3. Treatment nonadherence.
4. Unapproved/intentional/general use.

Four separate base-adjusted models and one mutually adjusted model were specified. Leave-one-preferred-term-out analyses reconstructed P1 after removing one frozen term at a time. Domain models were exploratory; no clinical homogeneity of the aggregate was assumed.

### 4. Reporting-process sensitivity

The expanded model added log(1+preferred-term count), log(1+drug count), literature source, and US origin to the core P1 model. These variables were treated as reporting-process proxies rather than patient-level confounders.

A P1-by-report-period model and calendar-period-specific estimates were specified. Annual observed reporting proportions were to be interpreted as reporting-process measures, not incidence trends.

### 5. Within-F1 management phenotype

Within F1 reports, M1-positive and M1-negative reports were compared descriptively. A Firth model for M1 included report period, healthcare-professional reporter, manufacturer origin, P1, anticoagulant co-report, and antiplatelet co-report where estimable.

### 6. Timing completeness and windows

Within F1, availability of a non-negative report-level timing interval was modeled with M1, P1, report period, healthcare-professional reporter, manufacturer origin, age availability, log(1+preferred-term count), and log(1+drug count).

Among date-complete reports, prespecified mutually exclusive intervals were 0–7, 8–14, 15–28, and >28 days. Cumulative proportions were calculated at 1, 7, 14, and 28 days with exact binomial 95% confidence intervals.

### 7. Age sensitivity

The core P1 model was repeated in reports with observed age and in all reports with missing age represented as an explicit category. These analyses were sensitivity analyses and were not intended to resolve informative missingness.

### 8. Decontaminated report-richness safeguard

After the first expanded model run, it was recognized that total preferred-term count mechanically contained outcome- and predictor-defining terms. The raw-count model was retained as specified. An additional decontaminated model was added in which F1, I1, and P1 defining terms were excluded from the preferred-term count. This safeguard and its timing were documented before final interpretation.

## Part II. Sequential augmentation locked on 13 July 2026

### 9. Restricted coding ladder and severity contrasts

Three mutually exclusive coding rungs were specified:

- L0: I1 without M1;
- L1: F1 without M1;
- L2: F1 with M1.

I1 with M1 was retained descriptively but excluded from the restricted ordering. Continuation-ratio contrasts, parallel overlapping severe-outcome contrasts, and within-F1 models were specified with the fixed core adjustment. Sparse cells were labeled and not treated as confirmatory.

### 10. One-way differential P1 under-recording analysis

The bias scenario assumed that P1 could be selectively missing from F1 reports while observed I1 coding remained fixed. For each integer k, P1-negative F1 reports were reclassified as positive. Thresholds were specified for:

- crude Fisher P≥0.05;
- adjusted 95% CI including 1;
- equal crude P1 proportions;
- adjusted point estimate ≥1.

Random allocation without replacement represented uncertainty about which covariate profiles were under-recorded. The final implementation used k=0–80, 1,000 repetitions per k, and seed 20260808. The analysis measures fragility and does not estimate the amount of missing coding.

### 11. Clinical evidence triangulation

A structured, non-pooled comparison was specified using the FDA prescribing information, pivotal or pooled clinical-development evidence, a chart-reviewed Veterans Affairs cohort, and the present FAERS phenotype. The manuscript position was fixed as refined characterization of a known serious risk.

### 12. Conditional same-indication comparator scan

An inferential comparator required a clinically coherent alternative therapy with at least 50 reports and at least five F1 events. Simple covariate adjustment additionally required at least 10 F1 events in each exposure group. Heterogeneous residual therapies were not to be pooled merely to reach a numerical threshold.

## Part III. Amendment A1 dated 13 July 2026

Amendment A1 was added after the initial ladder and P1 models and before the following analyses were fitted:

5. A categorical hospitalization model comparing L1 and L2 separately with L0.
6. A linear one-rung hospitalization trend.
7. A deterministic covariate-balanced P1 reclassification path using proportional allocation and largest-remainder integer apportionment across report-period, reporter, and manufacturer strata.
8. An increase from 500 to 1,000 random allocations per k to reduce Monte Carlo error.
9. A supportive crude two-sided Fisher non-significance threshold, distinguished from crude prevalence equality.

The hospitalization analyses were conceived as an internal check on the restricted ordering. In the manuscript, their interpretation is deliberately limited to supportive internal consistency and not clinical validation.

## Part IV. Peer-review clarification analyses dated 28 August 2026

The following descriptive or quality-assurance analyses were added in direct response to peer review and are labeled as such rather than prespecified confirmatory analyses:

10. Exact raw P1 proportions and two-sided Fisher exact crude OR were systematically juxtaposed with the secondary adjusted estimate in the main display.
11. The 2,379 AS1 reports meeting neither F1 nor I1 were characterized by calendar period, M1, hospitalization, and joint M1-plus-hospitalization coding.
12. All observed frozen F1, I1, M1, and P1 strings were audited against MedDRA version 29.0 to identify label transitions and unmapped observed concepts.
13. Counts were rechecked across all deletion-list-aware outputs, including F1 446→445 and F1+M1 195→194.

These additions refine transparency and interpretation; they do not redefine the cohort, phenotype rules, core model, or inferential boundaries.
